# Cellular hierarchy framework based on single-cell/multi-patient sample sequencing reveals metabolic biomarker *PYGL* as a therapeutic target for HNSCC

**DOI:** 10.1186/s13046-023-02734-w

**Published:** 2023-07-08

**Authors:** Jiezhong Guan, Xi Xu, Guo Qiu, Chong He, Xiaoyue Lu, Kang Wang, Xinyu Liu, Yuanyuan Li, Zihang Ling, Xuan Tang, Yujie Liang, Xiaoan Tao, Bin Cheng, Bo Yang

**Affiliations:** 1 Hospital of Stomatology, Guanghua School of Stomatology, Sun Yat-Sen University, Guangdong Provincial Key Laboratory of Stomatology, Guangzhou, China; 2 Department of Hematology, Nanfang Hospital, Southern Medical University, Guangzhou, China; 3 Zhongshan School of Medicine, Sun Yat-Sen University, Guangzhou, China

**Keywords:** HNSCC, Cellular hierarchy framework, Single-cell sequencing, Cell metabolism reprogramming, *PYGL*

## Abstract

**Background:**

A growing body of research has revealed the connection of metabolism reprogramming and tumor progression, yet how metabolism reprogramming affects inter-patient heterogeneity and prognosis in head and neck squamous cell carcinoma (HNSCC) still requires further explorations.

**Methods:**

A cellular hierarchy framework based on metabolic properties discrepancy, METArisk, was introduced to re-analyze the cellular composition from bulk transcriptomes of 486 patients through deconvolution utilizing single-cell reference profiles from 25 primary and 8 metastatic HNSCC sample integration of previous studies. Machine learning methods were used to identify the correlations between metabolism-related biomarkers and prognosis. The functions of the genes screened out in tumor progression, metastasis and chemotherapy resistance were validated in vitro by cellular functional experiments and in vivo by xenograft tumor mouse model.

**Results:**

Incorporating the cellular hierarchy composition and clinical properties, the METArisk phenotype divided multi-patient cohort into two classes, wherein poor prognosis of METArisk-high subgroup was associated with a particular cluster of malignant cells with significant activity of metabolism reprogramming enriched in metastatic single-cell samples. Subsequent analysis targeted for phenotype differences between the METArisk subgroups identified *PYGL* as a key metabolism-related biomarker that enhances malignancy and chemotherapy resistance by GSH/ROS/p53 pathway, leading to poor prognosis of HNSCC.

**Conclusion:**

*PYGL* was identified as a metabolism-related oncogenic biomarker that promotes HNSCC progression, metastasis and chemotherapy resistance though GSH/ROS/p53 pathway. Our study revealed the cellular hierarchy composition of HNSCC from the cell metabolism reprogramming perspective and may provide new inspirations and therapeutic targets for HNSCC in the future.

**Supplementary Information:**

The online version contains supplementary material available at 10.1186/s13046-023-02734-w.

## Background

Head and neck squamous cell carcinoma (HNSCC) is the most prevalent type of malignancy in the head and neck region with high incidences and adverse 5-year survival rate [1]. Poor clinical outcomes are attributed to extensive inter-patient heterogeneity and chemotherapy resistance, highlighting the inadequacy of standard therapy for curing patients with HNSCC [2]. Therefore, further exploration of cellular mechanisms that drive HNSCC development and identification of new therapeutic targets for overcoming chemotherapy resistance, are of great significance for improving the prognosis of HNSCC patients.

Previous studies have shown that cancer cells often perform epigenetic alteration to tackle challenges from microenvironment and promote adaptability [3]. Cell metabolism reprogramming, one of the crucial tumor epigenetic alterations in response to fluctuating energy needs [4], allows cancer cells to dynamically adjust energy generation and bioenergetics through several metabolic pathways such as glycolysis [5], gluconeogenesis [6], lipid metabolism [7] and arachidonic acid metabolism [8]. Malignant behaviors including proliferation, invasion and metastasis have been proved to correlate with cell metabolism reprogramming [9], wherein biological processes have been increasingly revealed. For instance, cancer cells can stimulate metastasis through targeting fatty acid receptor CD36 via O-GlcNAcylation [10, 11], or affect lipid metabolism through interferon-γ and granzyme B to alter the function of stromal and immune cells from microenvironment, leading to tumor invasion and recurrence [12]. Simultaneously, metabolic changes in cancer cells are also associated with chemotherapy resistance [13]. Activation of glutamine metabolism could significantly up-regulate adipogenesis, leading to sorafenib resistance in tumor [14]. Moreover, resistance of gemcitabine can be induced by increasing glycolysis mediated by hypoxia-inducible factor (HIF)-1α [15]. In allusion to chemotherapy resistance related cell metabolism reprogramming, studies of targeting metabolic changes through the metabolic core pathways in tumor cells has been emerging [16, 17], which may better solve the drug resistance of tumors and improve the prognosis of patients.

Existing studies have confirmed that the changes of metabolic characteristics of HNSCC cells through diverse metabolic pathways inducing the malignant behaviors and chemotherapy resistance [18–20], but the relationship between these alterations and patient prognosis has not yet been systematically elucidated. On the other hand, cell metabolism reprogramming may also contribute to the heterogeneity of HNSCC patients [21].Various research about the underlying mechanisms of metabolism reprogramming emerged recent years, indicating it would be a promising direction to break through. Single-cell RNA sequencing (scRNA-seq) has emerged as a powerful tool for dissecting cellular hierarchies [22], and there has been a study utilizing this technique on metabolic characteristics in cellular hierarchies of tumor cells [23]. However, owing to limited number of patients from scRNA-seq datasets, few studies have comprehensively explored the link between metabolism reprogramming reflected in cellular hierarchy composition and inter-patient heterogeneity, which may account for the poor prognosis and chemotherapy resistance in HNSCC. With integration of single-cell and multi-patient sample sequencing, we explored the relationship of specific tumor cell subsets and patient’s prognosis, and further revealed the therapeutic target for more information from the combination of sequencing data.

**Fig.1:**
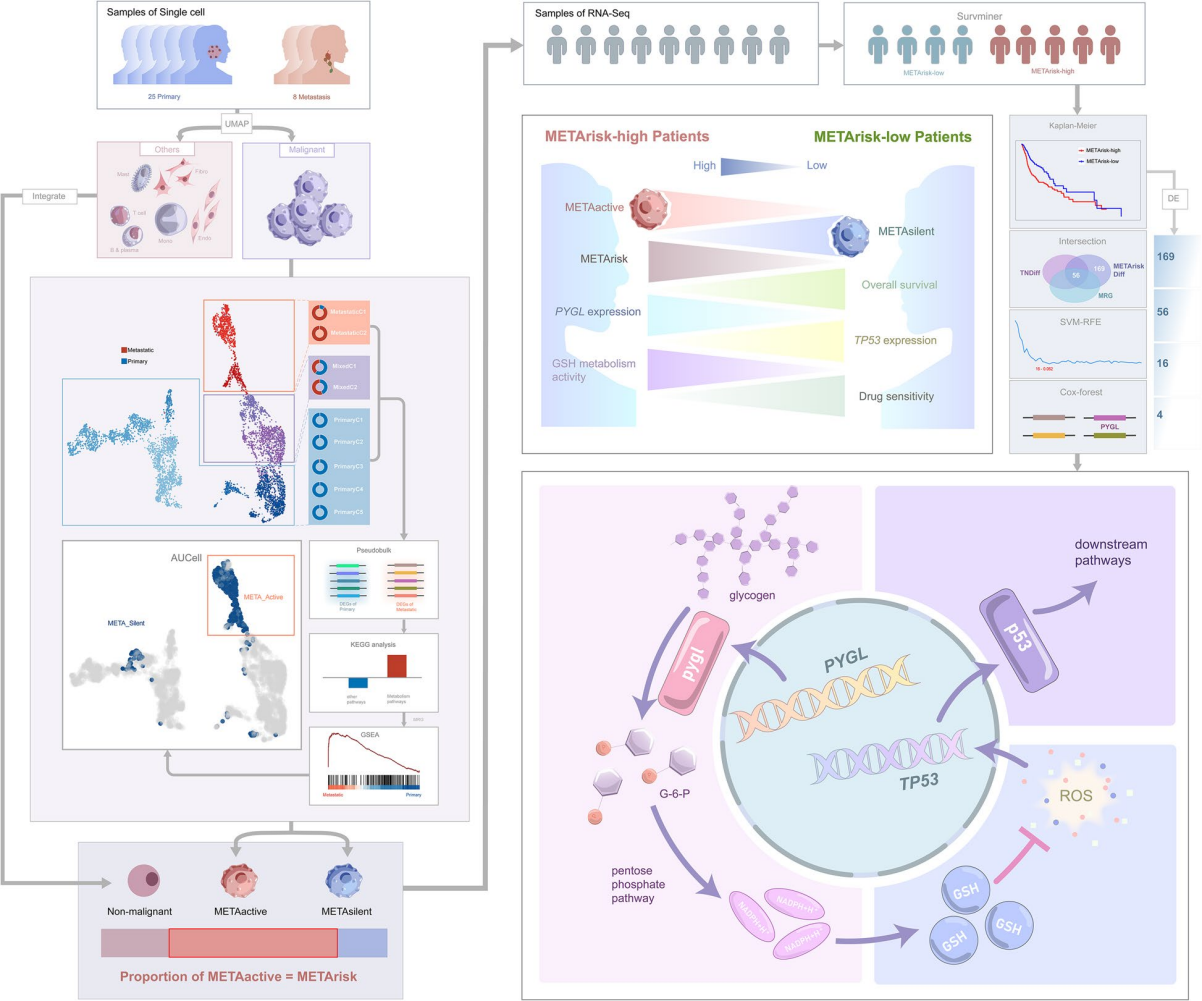
Overall design of the study

In this study, the cellular hierarchies of 486 patients with HNSCC were characterized through gene expression deconvolution on bulk HNSCC transcriptomes using single-cell reference profiles of distinct HNSCC cell types including METAactive and METAsilent malignant cells and integrated non-malignant cells from 25 primary and 8 metastatic HNSCC samples. METAactive cells were classified as the particular malignant cluster with high potential of cell metabolism reprogramming and metastasis and METAsilent cells possessed comparatively low metabolic activity and mainly distributed in primary tumor, by which patients were divided into METArisk-high and METArisk-low groups based on the proportion. Later, *PYGL* was identified as the key metabolism-related biomarker that drives the poor prognosis and drug resistance of patients in METArisk-high group, wherein *PYGL*/GSH/ROS/p53 was demonstrated as a key pathway (Fig. 1 and Fig.S1). Sequentially, our study may provide reference for further understanding of genetic characteristics of cancer metabolism of HNSCC, and how metabolism reprogramming affects tumor malignancy and chemotherapy resistance in HNSCC patients, thus, hopefully providing a new target in metabolic pathways to improve patient prognosis.

## Methods

### Data source

The single-cell RNA expression profile of 25 primary and 8 metastatic head and neck squamous carcinoma (HNSCC) samples were downloaded from NCBI Sequence Read Archive (SRA) with an accession #: SRP332116, SRP228811. The mRNA expression profile and clinical information of multi-patient HNSCC samples were downloaded from The Cancer Genome Atlas (TCGA) (http://portal.gdc.cancer.gov/) database and The Gene Expression Omnibus (GEO) (https://www.ncbi.nlm.nih.gov/geo/) database.

### Quality control of single-cell and bulk tissue transcriptomic samples

The inclusion criteria for the single-cell transcriptomic samples includes: (1) From the datasets ≥ 4 samples, at least 2 controls and 2 cases, and availability of raw data or gene expression data; (2) Extraction protocol:Droplet-based single-cell RNA-sequencing on 10X Genomics Chromium platform.

A standard Seurat quality control, transformation, and data integration was then performed: (1) the data with low-quality cells expressing ≤ 200 genes or ≥ 2500 genes or the percent of mitochondrial genes infinite or ≥ 15% were removed and the data with genes expressed in less than 3 cells were removed; (2) For better interpretability, the gene expression matrixes were then transformed using the 'ScaleData' function in Seurat with 3000 highly variable genes, regressions were applied to reduce the effects of mitochondrial genes; (3) 'FindIntegrationAnchors' and 'IntegrateData' function was then used to integrate datasets.

The inclusion criteria for the bulk tissue transcriptomic samples were: (1) Non-formalin soaking samples; (2) With complete clinical metadata; (3) With definite clinical outcome and survival time.

### Pre-processing of scRNA-sequencing data

CellRanger (version 4.0.0) was used to obtain the fastq files of the raw data and annotated with the human genome reference sequence (GRCh38). The gene-barcode matrix was then obtained following the Seurat (version 4.0.4) pipeline in R software (version 4.0.5, R-Foundation, Vienna, Austria).

### Cell clustering analysis, visualization, and annotation

Cell-clustering and sub-clustering analyses were performed with the FindClusters function of the Seurat package with proper resolutions. For the re-clustering of each type of cell clusters, cells with ribosome gene ratio higher than 35% were filtered. Uniform manifold approximation and projection (UMAP) was used to display identified cell clusters and sub-clusters. The cell clusters were annotated based on highly expressed genes, unique expressed genes, and reported canonical cellular markers.

### Clustering and cell type assignment

First, a neighborhood graph was constructed to identify related groups of cells. Next, Leiden clustering was performed on the neighborhood graph. The cluster assignments were then visualized on UMAP plots. Using a combination of top expressed genes in each cluster and a list of known marker genes, cell types were assigned to each cluster.

### Pseudobulk

Pseudobulk was used to detect the Differential expressed genes (DEGs) between primary- and metastatic- specific populations, for it aggregates count values from each sample and cell cluster to create data that can be analyzed using the same methods as bulk RNA-seq data, maintaining the same number of genes but reducing the number of cells to the number of samples in the gene expression matrix [24].

### Functional analysis

The DEGs for the malignant group were independently imported into Enrichr, an online bioinformatic website (https://maayanlab.cloud/Enrichr/) for Kyoto Encyclopedia of Genes and Genomes (KEGG) analysis. The top 10 pathways were selected based on p-value ranking. “META_ACTIVATE” was defined as the gene signature from metabolism pathways selected in KEGG analysis intersected with genes from single cell database. Differential expression rank order was used for subsequent Gene Set Enrichment Analysis (GSEA), performed using the clusterProfiler package in R.

### AUCell

META_ACTIVATE gene signature was set as input data for area under the curve (AUC) value calculation. According to the AUC value, gene-expression rankings were built for each cell. The AUC estimates the proportion of genes in the gene set that are highly expressed in each cell. Cells expressing more genes from the gene set will have higher AUC values than cells expressing fewer genes. Function “AUCell_exploreThresholds” was used to calculate the threshold that could be used to consider the current gene-set active. Then, cell clustering UMAP embedding was colored based on the AUC score of each cell to show which cell clusters were active in the META_ACTIVATE gene signature. Metabolism-related genes (MRGs) for downstream analysis were obtained from the intersection of the META_ACTIVATE gene signature and cell cluster with high AUC score.

### CIBERSORTx

Raw gene expression counts from single cell database were used as input for signature matrix generation with CIBERSORTx28. Default settings were used, with the exception of the minimum expression parameter, which was set to 0.25. Deconvolution was performed on normalized Transcripts per million (TPM) bulk RNA-seq data using S-mode batch correction and Absolute mode.

### Analysis of the prognostic and therapeutic value of MRGs in HNSCC

“Limma” R package was used to identify DEGs between 486 HNSCC and 44 normal samples and between METArisk-low and METArisk-high group with |log_2_FC|> 1 and adjusted *p*-value < 0.05. Then the DEGs were overlapped with MRGs to obtain Differential Expressed Metabolism-Related Genes (DEMRGs) involved in HNSCC. A customization of an open-source the support vector machine recursive feature elimination (SVM-RFE) R script (https://github.com/johncolby/SVM-RFE) based on the R package “e1071” was made to train samples through the training set, sorting the scores of DEMRGs and estimation of tenfold cross-validation errors of gene combinations. The gene combination with the least tenfold cross-validation errors was selected as SVM-RFE generated prognosis-related gene candidates. Multivariate Cox regression analysis was used to identify the independent prognostic factors among the DEMRG candidates and the prognostic value was evaluated by Kaplan–Meier analysis. Images of immunohistochemistry (IHC) staining of the protein products of the genes in HNSCC samples were extracted from the Human Protein Atlas (HPA) database (http://www.proteinatlas.org). Computational Analysis of Resistance (CARE) is a software for identifying genome-scale biomarkers of targeted therapy response using compound screen data. “OncoPredict” is an R package for predicting the drug response, by which the associations of gene expression with the sensitivities to the commonly utilized chemotherapy drugs were investigated.

### Metabolic heterogeneity analysis of *PYGL*-low and *PYGL*-high groups

To characterize the metabolic features of *PYGL*-low and *PYGL*-high groups, the enrichment of 113 metabolism processes in different clusters were quantified by “GSVA” R package and the differential analysis was conducted by “limma” R package. Thereafter, unsupervised non-negative matrix factorization (NMF) clustering was performed using “NMF” R package with the settings “nrun = 50, method = brunet” based on the genes involved in the metabolism process “GSH metabolism”. According to the maximum of cophenetic and dispersion values in rank survey, HNSCC patients were divided into glutathione (GSH) metabolism up-regulated and GSH metabolism down-regulated clusters. To characterize the of different clusters, we analyzed and compared the Estimation of Stromal and Immune cells in Malignant Tumor tissues using Expression (ESTIMATE) score and tumor purity score calculated by the ESTIMATE algorithm. The survival among two clusters were analyzed by Kaplan–Meier curves using “survminer” R package.

### Cell culture and cell transfection

HNSCC cell lines CAL-27 (RRID: CVCL_1107) and HSC-6 (RRID: CVCL_A615) were cultured in Dulbecco's modified Eagle's medium (DMEM, Invitrogen, Grand Island, NY, USA) with 10% fetal bovine serum (FBS, Sigma-Aldrich) at 37℃ in an incubator with 5% CO_2_. For cell transfection experiments, CAL-27 and HSC-6 cells were seeded in 24-well plates for 24 h with Opti-medium before transfection. The cells were treated with siRNA using lipofectamine (ThermoFisher, China) according to the manufacturer’s instructions after growth to 70–80% puromycin (Sellek, USA) was used to select the stable transfected cells.

### Patient samples

Paired tumor and peritumor tissues were collected from HNSCC patients. Peritumor samples were collected 2–5 cm from the edge of tumor tissue. All patients were surgically proven HNSCC, and the follow-up was performed by the Hospital of Stomatology, Guanghua School of Stomatology, Sun Yat-sen University. Samples were obtained with informed consent, and the study was approved by the Institutional Ethics Review Board.

### Cell proliferation assay

The Cell Counting Kit-8 (CCK-8, Dojindo, China) was used to measure proliferation ability of CAL-27 and HSC-6 cells. The cells were cultured in a 96-well plate after transfection. 100μL Opti-medium and 10ul CCK-8 solution were added to each well at 24, 48 and 72 h time points. After 30 min of incubation, the optical density (OD) value at 450 nm of each well was evaluated by microplate reader machine (BioTek, Vermont, USA). Experiments were repeated at least three times.

### Transwell assay

Cell migration and invasion were evaluated by Transwell assay. For cell migration experiments, CAL-27 and HSC-6 cells were seeded in a 24-well plate after transfection. 300ul DMEM with 10% FBS was added to the 24-well plate in prior and 200ul cell suspension was added to the upper chambers containing serum-free medium after 1 h. Photographs of non-overlapping fields were taken after 18 h cell incubation in the present of 5% CO2 at 37℃. For cell migration experiments, 300ul DMEM with 10% FBS was added in the 24-well plate while Matrigel matrix (CORNING, USA) was added to the center of upper chamber at a concentration of 50ul/cm2 growth area. After Matrigel formed into a gel, 200ul cell suspension was added to the upper chambers containing serum-free medium. Photographs of non-overlapping fields were taken after 24 h cell incubation in the present of 5% CO2 at 37℃. Experiments were repeated at least three times.

### Flow cytometry assay

CAL-27 and HSC-6 cells were washed with and resuspended in phosphate buffered solution. 7-aminoactinomycin D (7-AAD) (A1310, Life technologies) was used to exclude dead cells. For cell cycle analysis, cells were fixed with 4% paraformaldehyde (MA0192, Meilunbio) for 12 h at 4℃, permeabilized with 0.2% Triton X-100 (T9284, Sigma) in PBS for 15 min at room temperature, and then stained by antibodies to Ki67 (16A8, Biolegend) and further incubated with 0.1 μg μL-1 4’,6-diamidino-2-phenylindole (DAPI, 1306, Thermo Scientific) for 30 min at room temperature. For apoptosis analysis, cells were stained by AnnexinV (640,907, Biolegend) and 7-AAD. For intracellular reactive oxygen species (ROS) levels, cells were stained with antibodies to LSC phenotypic markers, and further stained by 5 μM Dihydroethidium (DHE, KGAF019, KeyGEN) for 30 min at 37 °C. For glucose uptake assay, glucose uptake was measured using the fluorescence-labeled deoxyglucose analog 2-(N-(7-nitrobenz-2-oxa-1,3-diazol-4-yl) amino)-2-deoxyglucose (2-NBDG, Apex Bio, USA). After the indicated treatment, cells were incubated with 2-NBDG for 1 h at 37 °C. Cell sorting and analysis were performed using either a flow cytometer (Attune NxT; Thermo Fisher) or sorter (InFlux Cell or FACSARIA III, BD Biosciences). Data analysis was performed using FlowJo software.

### Immunofluorescence assay

Cells were incubated for 3 h with a Histone Rabbit mAb (Cell signal, #7631S). Incubations were performed at room temperature. The slides were collected and manually washed three times with Ventana buffer and three times with distilled water. Finally, the slides were counterstained for 2 min with 1ug/ml of 4,6-diamidino-2-phenylindole (#D1306, Life Technologies, Oregon, USA). γ-H2AX staining were used to indicate double-stranded DNA damage and apoptosis [25]. A proprietary image analysis system was used to quantify the number of positive cells over the total number of cells and the average signal emission/nucleus.

### Western blot

Total protein was collected from CAL-27 and HSC-6 cells using RIPA lysis buffer (Beyotime, Shanghai, China), separated by 10% sodium dodecyl sulphate–polyacrylamide gel electrophoresis (SDS-PAGE) gels and then transferred to polyvinylidene fluoride (PVDF, Millipore) membranes. The proteins were incubated with primary antibodies overnight at 4 °C, and then incubated with secondary antibodies for 1 h at room temperature. An Enhanced Chemiluminescence (ECL) Detection Kit (Millipore) was used to visualize the immunoreactive proteins. The information of antibodies: p53 (1:1000); p21 (1:1000). The antibodies against *PYGL* (Abcam, ab227403), p53 (Cell signaling, #2524S), p21 (Cell Signaling, #2947S) were used for western blotting.

### RNA extraction and qRT-PCR

TRIzol (Invitrogen) was used to extract total RNA from HSC-6 and CAL-27 cells and tissue samples according to the manufacturer’s instructions. The concentration and purity of total RNA were examined by UV spectrophotometric analysis at 260 nm. The cDNAs were synthesized using a reverse transcription kit (Promega, Madison, WI, USA) according to the manufacturer's instructions. The mRNA expression levels were measured using the SYBR Green PCR Master Mix (Applied Biosystems, Waltham, MA, USA) and the Applied Biosystems 7900HT sequence detection system (Applied Biosystems). And mRNA relative expression level was measured using the 2 − ΔΔCt method and normalized to glyceraldehyde 3-phosphate dehydrogenase (GAPDH).

### PAS staining and glycogen assay

Glycogen level was measured using standardized periodic acid Schiff (PAS) staining technique by AB-PAS staining kit (Bioss). Cells were fixed with 4% paraformaldehyde for 15 min, incubated in 1% periodic acid for 5 min, rinsed in water, and placed in Schiff’s reagent for 10 min. Finally, cells were washed with water. Amylase was used in a set of experiments to verify that staining was specific for glycogen.

### Detection of ROS

ROS levels were determined using MitoSOX Red (Applygen, China) as an indicator of mitochondrial superoxide. CAL-27 and HSC6 cells were treated with MitoSOX Red according to the instruction by manufacturer after transfection for 24 h. Cells were washed using PBS and analyzed by FACSCantoTM (BD Biosciences). Data analysis was performed using FlowJo software (Tree Star, Ashland, OR). Experiments were repeated at least three times.

### Detection of GSH

GSH levels were determined using a colorimetric GSH assay kit according to the manufacturer’s instruction. Briefly, cells (10 µg) were mixed with 30 µl 5% metaphosphoric acid, and then frozen and thawed twice using liquid nitrogen and 37 °C water. The samples were centrifuged, and the supernatant was subjected to a GSH assay based on a kinetic enzymatic recycling method that detects the oxidation of GSH by 5,5′-dithiobis-2-nitrobenzoic acid (DTNB) and GSH reductase to measure the GSH content in cells74. The absorbance was measured at 412 nm with the Microplate Readers.

### Xenograft tumor model

Female BALB/c nude mice (RRID: IMSR_JCL:JCL:mID-0001, 4-weeks-old, purchased from Gempharmatech-GD, Guangdong, China) were maintained in a specific pathogen-free room in the sterile animal facility. *PYGL* knock-down and control cells were cultured in 100 mm culture dishes and collected by trypsin. The cells were injected subcutaneously into the submandibular region of BALB/c nude mice at a density of 10^6^ in 100 μl medium. The mice were randomly divided into four groups: for the *PYGL* KD group, the mice were injected with *PYGL* KD tumor cells. For the cisplatin chemotherapy group, mice were administered injection of cisplatin (0.2 ml/20 g/d) and NaCl (0.4 ml/20 g/d), The control mice were administered the same volume of NaCl (0.6 ml/20 g/d). Tumor volumes were measured twice a day and calculated according to the following formula: 0.5 × length × width^2^. The lung tissue was harvested for sectioning and slice culture to evaluate tumor metastases. All the section images were evaluated by two qualified pathologists in the Department of Pathology of the Hospital of Stomatology, Guanghua School of Stomatology, Sun Yat-sen University. When the results reported by the two pathologists were found to be consistent, they were considered as the verified outcomes. However, in case of any inconsistencies, a third pathologist from the Department of Pathology would reevaluate the results. All the pathologists had obtained the Certificate of Licensed Practicing Physician (Medical examination and Pathology). Mice were euthanized after 60 days of observation. Our experimental procedures were approved by the Institutional Ethics Review Board.

### Statistical analyses

Statistical analyses were performed with R (version 4.2.1) and GraphPad Prism 9.2. For comparisons between two groups, statistical significance was estimated using Wilcoxon test. For comparisons among three groups, Kruskal–Wallis test was applied. Pearson correlation analysis was used to determine the correlation between two variables. Survival analysis was performed by using a log-rank test. A p-value < 0.05 was considered statistically significant unless specified.

## Results

### Heterogeneity among HNSCC driven malignant metastasis was related to cell metabolism reprogramming

**Fig.2:**
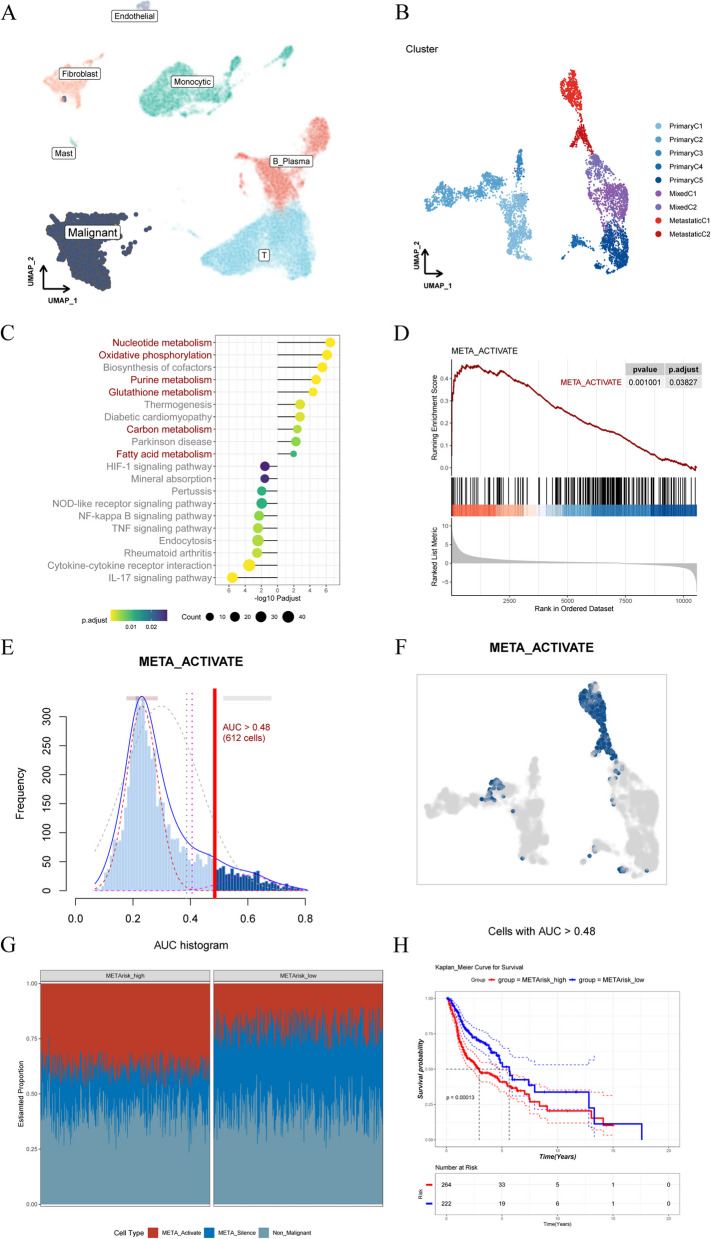
Cellular populations of HNSCC correlated with metabolic properties and survival. **A** Diffusion map of cells from all sc-RNA samples, colored by cell-type annotation. **B** Diffusion map of malignant cells colored by cell proportion annotation, including 9 clusters. **C** KEGG pathway enrichment analysis of DEGs between primary- and metastatic-specific populations. **D** GSEA analysis of “META_ACTIVATE” gene signatures between primary- and metastatic-specific populations, which were at the right and left side respectively. **E** AUC score of all cells from HNSCC samples. The threshold was marked by red bar and the cells colored by darked blue exceeded the threshold value. **F** Diffusion map based on the AUC score with cells exceeded the threshold value marked. **G** Relative abundance of different cell populations by gene expression deconvolution in each patient from METArisk-high and METArisk-low groups. Each bar represents an individual patient, and the distribution of colors throughout each bar represents the distribution of cells. **H** Kaplan–Meier analysis for survival probability of the two METArisk groups

As a first step to uncover the organization of cellular hierarchies in HNSCC, we re-analyzed the scRNA-seq profiles covering 25 primary and 8 metastatic HNSCC samples, and notably classified malignant cells, endothelial cells, fibroblast cells, monocytic cells, mast cells, T cells, and B/plasma cells (Fig. 2A, S2A and B). With a focus on the malignant population mainly triggering invasion and pathological state, malignant cells were extracted and re-clustered, while sample source was calculated for cluster identification. 5 clusters with high ratio of cells from primary cancer were named primary C1-C5, 2 clusters with abundant proportion of cells from metastatic tissue were named metastatic C1-C2, and the remain 2 clusters with hybrid sample origin were categorized as Mixed C1-C2 (Fig. 2B, S3A and B). Markers of different clusters were shown in Fig.S3C. The identification of cell clusters with different constitution revealed the existence of distinct cellular distribution in malignant cells, which provided a molecular basis for the known heterogeneity found within the malignant cells compartment [26]. Because metastatic clusters have more proportion of cells from metastatic tissue while primary clusters possess high ratio of cells from primary cancer, studying the heterogeneity between these two populations would provide cellular traits of tumor metastasis, which was considered as the behavior of later tumor period and associated with worse prognosis. Therefore, mixed clusters were discarded while characteristics of primary- and metastatic-specific populations were compared in the subsequent analysis. Using pseudobulk, a method for DEGs detection that avoids the interfere of several over-expressed genes and therefore reduces the false positives [24], we obtained the DEGs between two specific populations (Fig.S4).

Functional analysis based on pathway information from KEGG database illustrated that DEGs of metastasis-specific population were particularly enriched in several metabolism-related pathways including nucleotide metabolism, oxidative phosphorylation, purine metabolism, carbon metabolism and fatty acid metabolism (Fig. 2C), which indicated that cell metabolism reprogramming was supposed to be a magnitude explanation of HNSCC metastasis. Consequently, we intersected genes from KEGG metabolism-related pathways and genes expressed in HNSCC scRNA-seq profiles generated a set of genes named “META_ACTIVATE”. To investigate whether metabolism reprogramming was dynamic in metastasis population, GSEA was applied and the result showed that activity of most genes was inclined to activate in the metastasis-specific population (*P.adjust* = 0.038) (Fig. 2D). To quantify the metabolism activity in each cell, AUCell analysis, an efficient approach to identify cells with the META_ACTIVATE gene signature by evaluated threshold, was conducted, and we defined the cell with AUCell score exceeded than the threshold calculated as “META- active” cells, while other cells were defined as “META-silent” cells (Fig. 2E). The distribution of AUCell score was markedly hoisted and surpassed the threshold in metastatic C1-C2 among all of the malignant clusters, while AUCell score was inferior in other clusters **(**2F and S5A). The result represented that META-active cell significantly associated with metastasis, indicating that specific cell population could virtually influence the development of HNSCC. Additionally, 858 MRGs were screened out form the intersection between top marker genes of the “META-active” cells and META_ACTIVATE gene signature, which might serve as the potential metabolic biomarkers associated with tumor progression.

Meanwhile, expression profile of non-malignant cells from HNSCC microenvironment were integrated according to method depicted in the previous research on metabolic landscape of tumor microenvironment [23] for the downstream analyses.

### Deconvolution of cell populations in HNSCC revealed active metabolic properties leading to poor clinical outcomes

We next sought to figure out how these defined HNSCC cell populations and the hierarchies which they are organized into relate to functional, biological and clinical properties of HNSCC. Single-cell RNA sequencing analysis described the biological pattern provided the expression profile of META-active cell, META-silent cell and non-malignance cell, which enabled us to perform CIBERSORTx [27], a scRNA-seq-based gene expression deconvolution method to infer the cellular hierarchy composition in bulk HNSCC transcriptomes as 486 patient samples from TCGA database to examine how the metabolic properties vary across patient samples and how they relate to molecular and clinical features of HNSCC at scale in large clinical datasets (Fig. 2G). Obviously, we revealed the significant heterogeneity of cellular hierarchy composition among patients. Termed as “METArisk”, the proportion of META-active cells in each sample was calculated to describe the heterogeneity of cellular hierarchy composition of each patient and two subgroups were identified according to the segmentation value generated by optimal cutoff analysis: the “METArisk-high” group with higher METArisk and the “METArisk-low” group with lower METArisk (Fig.S5B). Kaplan–Meier analysis showed remarkable difference in prognosis between METArisk-high and METArisk-low group, wherein METArisk-high was associated with worse prognosis and METArisk-low was associated with better prognosis (Fig. 2H), suggesting that exploring the heterogeneity of METArisk group would contribute to finding specific biochemical characteristic lead to poor prognosis.

### *PYGL* was identified as a significant metabolic biomarker provoking high METArisk and poor prognosis.

**Fig.3:**
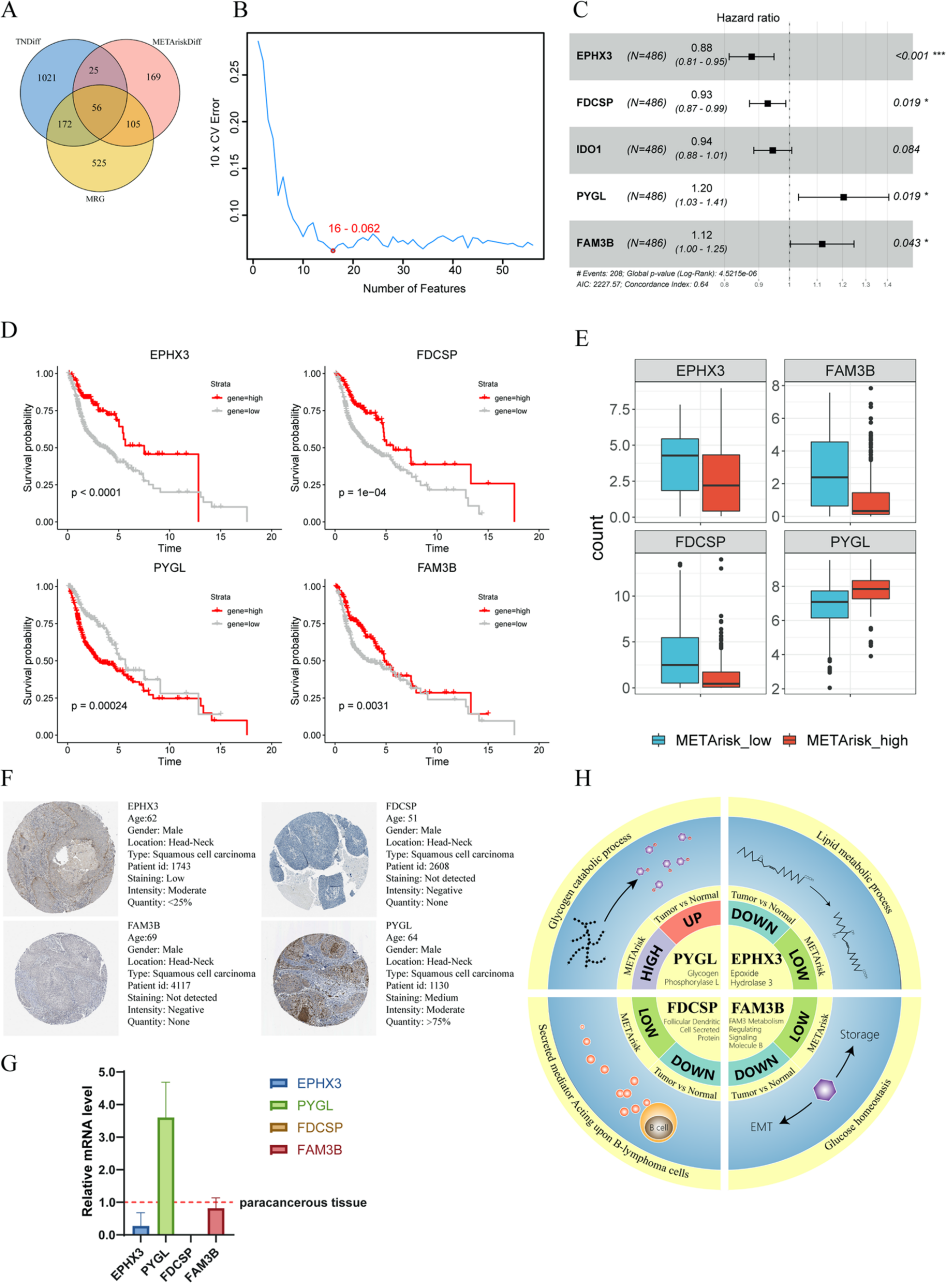
Identification of *PYGL* as a significant metabolic biomarker. **A** Venn diagram among METArisk-related DEGs, tumor-related DEGs and MRGs. **B** SVM-RFE used to select features having best discriminative capability for patient’s prognosis of HNSCC. **C** Multivariate Cox regression analyses discovered DEGs that can serve as independent predictors of clinical outcomes. **D** Kaplan–Meier survival curves of the four genes selected above. **E** Boxplot depicted the expression level of the four genes in METArisk-high and METArisk-low groups. **F** Representative IHC images of the four genes from the HPA database. **G** qRT-PCR analysis of the four genes. Relative mRNA expression level of tumor tissue compared with paracancerous tissure were exhibited in bar chart (*n* = 3). **H** The summary image of the four genes with research findings along with the biological functions

To further explore the underlying mechanisms for the observed association of METArisk and clinical outcomes of HNSCC, 56 candidate biomarkers were screened out after the intersection was taken using 858 MRGs of the META_ACTIVATE gene signature, DEGs between the two METArisk groups and those between tumor and normal samples (Fig. 3A). In consideration of the link between prognosis and gene expression, machine learning was employed to select biomarker that was closely correlated with the patient survival from the 56 candidates. To select combination of machine learning methods with the best prediction ability, we counted C-index and finally chose SVM-RFE and Mutivariate Cox PH regression analysis (Fig.S6). SVM-RFE first screened 16 genes related with overall survival (Fig. 3B), and multivariate Cox PH regression analyses identified four genes independently associated with clinical risk evaluated by both the outcomes and the survival time, including *EPHX3, FDCSP, FAM3B* and *PYGL* (*P* < 0.05, and *IDO1* was abandoned for *P* > 0.05) (Fig. 3C). To choose the metabolic biomarkers for intensive study, several analyses were conducted subsequently. Kaplan–Meier analysis showed that high expression of these genes led to better prognosis but for PYGL, which worsened the prognosis (Fig. 3D). Calculating the expression of four genes, *PYGL* was highly expressed in METArisk-high and tumor groups, while the others were highly expressed in METArisk-low and normal groups (Fig. 3E and S7). The different protein expression level of the four genes was detected by obtaining IHC staining image from the HPA database, which demonstrated that *PYGL* was highly expressed in HNSCC at translation level while the other was almost not expressed (Fig. 3F). RT-PCR experiment was conducted to verify the different expression of the four genes, which also exhibited the same variation (Fig. 3G). Our research findings along with the biological functions of the four metabolic biomarkers were summarized in Fig. 3H. Collectively, *PYGL*, the key element of the glycogen degradation process, was finally chose as the core biomarker of our research, for it was connected with high METArisk, led to poor prognosis, and was increasingly expressed in tumor at both transcriptional and translational level of HNSCC samples, which might sever as the driving factor for tumor-promoting cell metabolism reprogramming.

### *PYGL* was validated to stimulate HNSCC malignant behaviors and chemotherapy resistance.

**Fig.4:**
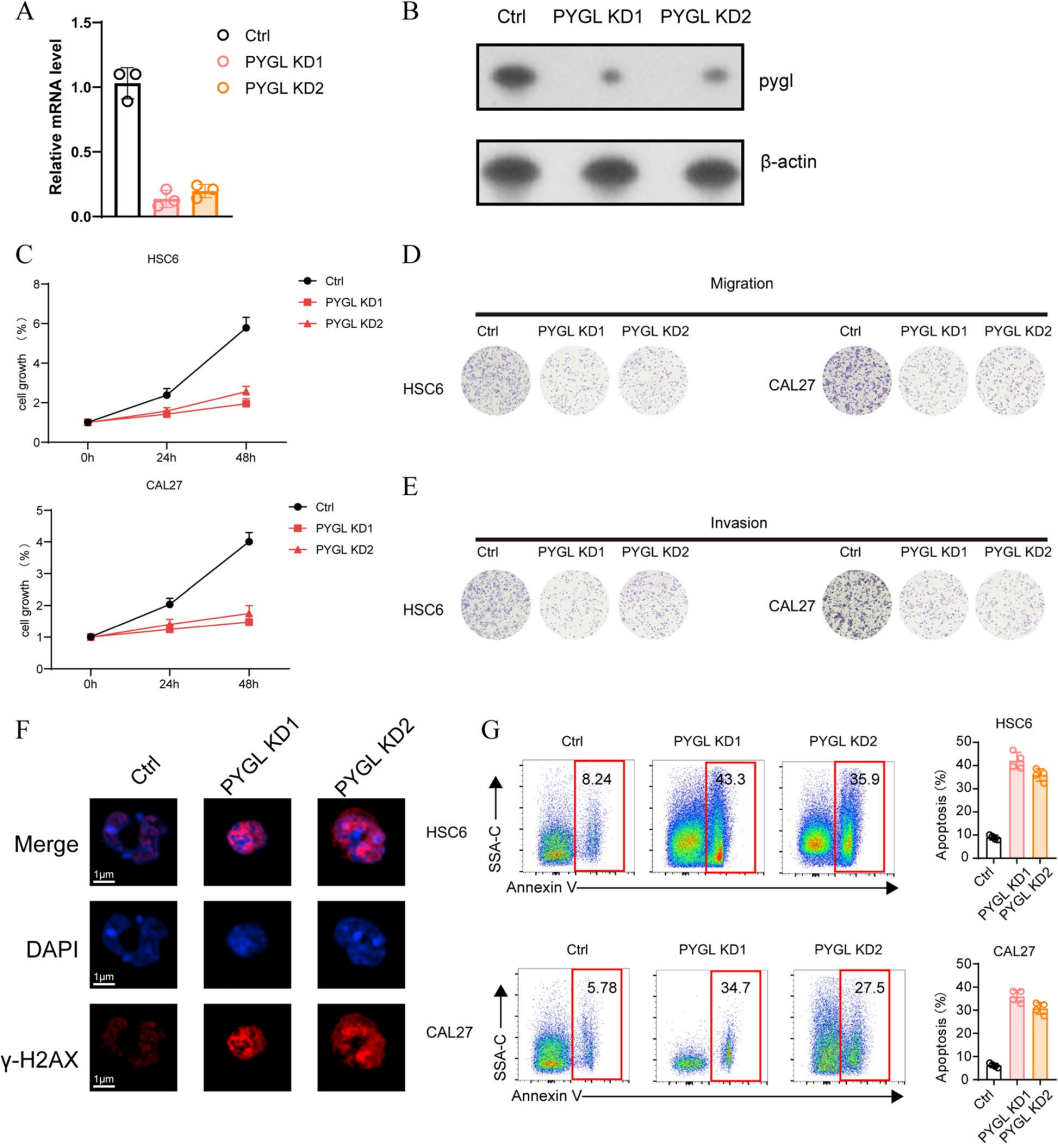
HNSCC malignant behaviors suppressed after *PYGL* knock-down. **A** and **B** qRT-PCR (**A**) and WB (**B**) analysis of the transfaction efficiency (*n* = 3 for independent experiments). **C** CCK-8 assay were used to detect cell proliferation capacities (*n* = 3). **D** and **E** Transwell assay were performed to examine cell potential of migration (**D**) and invasion (**E**). **F** Immunofluorescence staining showed the DNA damage (stained by γ-H2AX, red) in cells. Nuclei were counterstained with DAPI (blue) (*n* = 3). **G** Cell apoptosis was measured by flow cytometry assay. Cell was stained by AnnexinV and SSA-C and apoptotic cell was emphasized (*n* = 4)

To further explore the role of *PYGL* in HNSCC occurrence and development, we knocked down *PYGL* in HNSCC cell lines (HSC6 and CAL27) and verified the efficiency of transfection (Fig. 4A and B), and subsequently conducted a series of cell functional experiments. In cell proliferation assay, *PYGL* knock-down significantly suppressed HNSCC cell lines’ trend growth rate (Fig. 4C). In Transwell assay, migration (Fig. 4D) and invasion (Fig. 4E) of HNSCC cell lines was distinctly inhibited after *PYGL* knock-down. Immunofluorescence assay revealed that DNA damage occurred more frequently in *PYGL* knock-down group (Fig. 4F), and cell apoptosis analysis exposed that *PYGL* knock-down increased cell apoptosis rate (Fig. 4G). As the cellular alterations demonstrated in vitro, the expression of *PYGL* functionally promotes the malignant behavior of HNSCC, including promoting its growth, metastasis, invasion, and maintaining DNA integrity.

**Fig.5:**
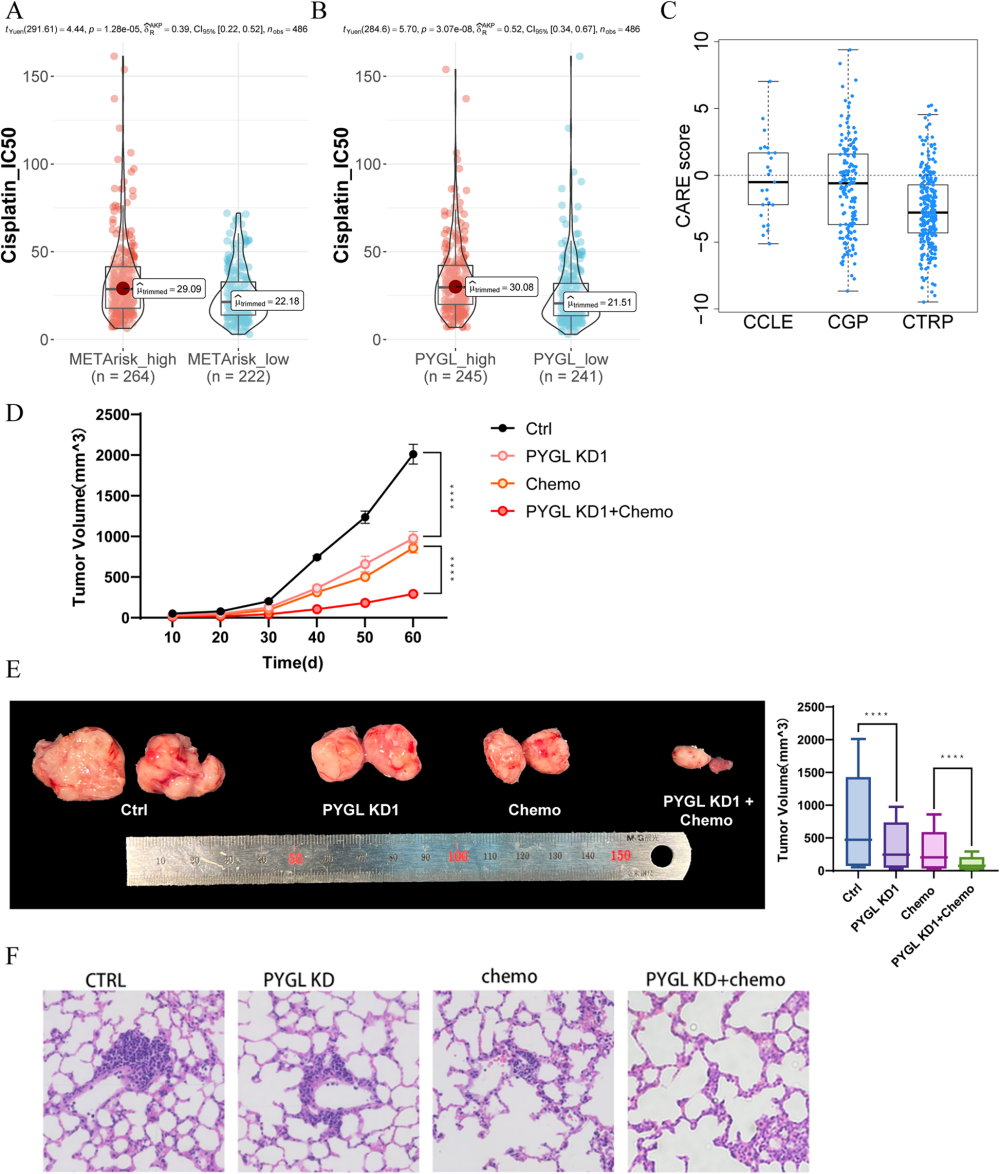
Chemotherapy resistance and metastasis of HNSCC promoted by *PYGL*. **A** Prediction of cisplatin IC50 in METArisk-high and METArisk-low groups. **B** Prediction of cisplatin IC50 in high and low *PYGL* expression groups. **C** Computational analysis of resistance with *PYGL* of cisplatin in the CARE database. **D** Tumor growth was measured once a week in tumor-bearing nude mice stably transfected with HNSCC cells (*n* = 5). **E** The tumors were removed, and the tumor weight was analyzed after the mice were executed. **F** Metastasis of HNSCC was measured by the results of hematoxylin–eosin staining of mice lung tissue

In addition to malignant behaviors, we also investigated the relationship between *PYGL* and chemotherapy resistance, another risk factor leading to poor prognosis in HNSCC. In accordance with ASCO Clinical Practice Guideline [28] and EHNS-ESMO-ESTRO Clinical Practice Guidelines [29], cisplatin was the first choice of chemotherapy to HNSCC, and consequently we implemented “Oncopredict” R package to predict IC50 of cisplatin in different groups. The METArisk-high group, with higher cellular hierarchy composition of META-active cells and up-regulated expression of *PYGL*, was correlated with higher IC50 of cisplatin (Fig. 5A). Splitting the HNSCC patients into subgroups according to the expression level of *PYGL*, higher IC50 of cisplatin was also observed in the *PYGL*-high group (Fig. 5B), and computer analysis showed a correlation with *PYGL* and drug resistance in the CARE database (CARE score < 0 in CCLE, CGP, CTRP respectively) (Fig. 5C), which indicated expression of *PYGL* was potential to induce chemotherapy resistance of cisplatin. Established by subcutaneously injecting HNSCC cell lines with *PYGL* knocked down into female BALB/c nude mice, xenograft tumor model was applied to validate the function of *PYGL* to cisplatin resistance of HNSCC. As Fig. 5D and E revealed, xenograft derived from *PYGL* knock-down cell lines was significantly diminished compared with the control, and cisplatin’s inhibition to HNSCC was also amplified at *PYGL* knock-down group. Furthermore, the effect of *PYGL* on HNSCC’s metastasis and its suppression by cisplatin was examined by determining the metastatic nodules in the lungs 60 days after inoculation, which illustrated that size of pulmonary metastatic nodules was suppressed while cisplatin’s function on metastasis suppression was increased in *PYGL* knock-down groups (Fig. 5F). In line with the cell function experiments in vitro, we demonstrated that the expression of *PYGL* was closely associated with HNSCC’s growth and metastasis in vivo, and further revealed its relationship with cisplatin’s resistance in the chemotherapy of HNSCC.

### *TP53*’s suppression was involved in *PYGL*’s promoting HNSCC malignancy

**Fig.6:**
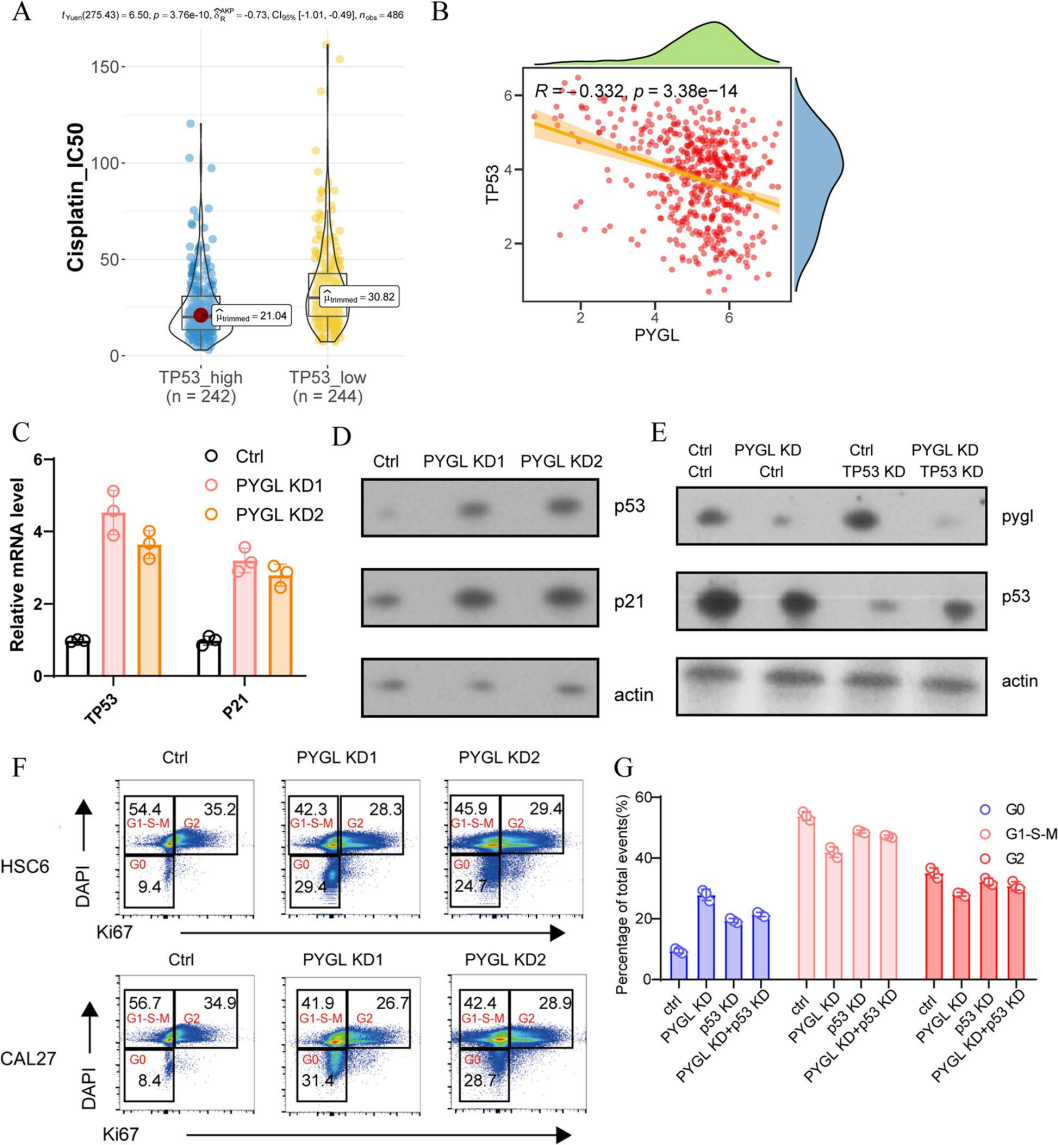
*PYGL* promoted HNSCC malignancy by suppressing *TP53* expression. **A** Prediction of cisplatin IC50 in high and low *TP53* expression groups. **B** Pearson correlation analysis between *PYGL* and *TP53* expression. **C** qRT-PCR of *TP53* and *P21* in *PYGL* knock-down groups (*n* = 3). **D** Western blot of p53 and p21 in *PYGL* knock-down groups. **E** Western blot of pygl and p53 in *PYGL* and *TP53* knock-down groups. **F** Flow cytometry using DAPI and Ki67 for cell cycle phase ratio analysis in *PYGL* knock-down groups (*n* = 3). **G** Statistics of cell cycle phase ratios analyzed in Fig. 5F

As previous studies have already proved that suppression of *TP53* was closely related with tumor progression, metastasis and chemotherapy resistance [30, 31], we also discovered that low *TP53* expression was associated with higher IC50 of cisplatin (Fig. 6A) and *TP53* had negative correlation with *PYGL* at transcriptional level (*R* = -0.332, *P* = 3.38e-14) (Fig. 6B), which drew to a speculation that *PYGL* might promote HNSCC’s chemotherapy resistance by suppressing *TP53*’s expression. To further ascertain the regulatory relationship between *PYGL* and *TP53*, cell experiment in vitro was performed to demonstrate that *PYGL* knock-down activated the expression of *TP53* along with its downstream target gene *P21* (Fig. 6C and D). Simultaneously, *TP53*’s expression obviously increased after knock-down employed on both *PYGL* and *TP53* (Fig. 6E), which indicated that *TP53* was on the downstream of *PYGL*’s regulation pathway. Moreover, flow cytometry assay detected that *PYGL* knock-down facilitated *TP53*’s function of cell cycle checkpoint on proliferative malignant cells in HNSCC, evidenced by the significantly increasing proportion of cell stagnated in G0 stage (Fig. 6F and G). Basically, our findings verified the expression of *PYGL* suppressed *TP53*’s expression and function, leading to abnormal proliferation and chemotherapy resistance in HNSCC.

### Glycogen degradation and GSH metabolism participated in *PYGL*’s promotion of HNSCC.

**Fig.7:**
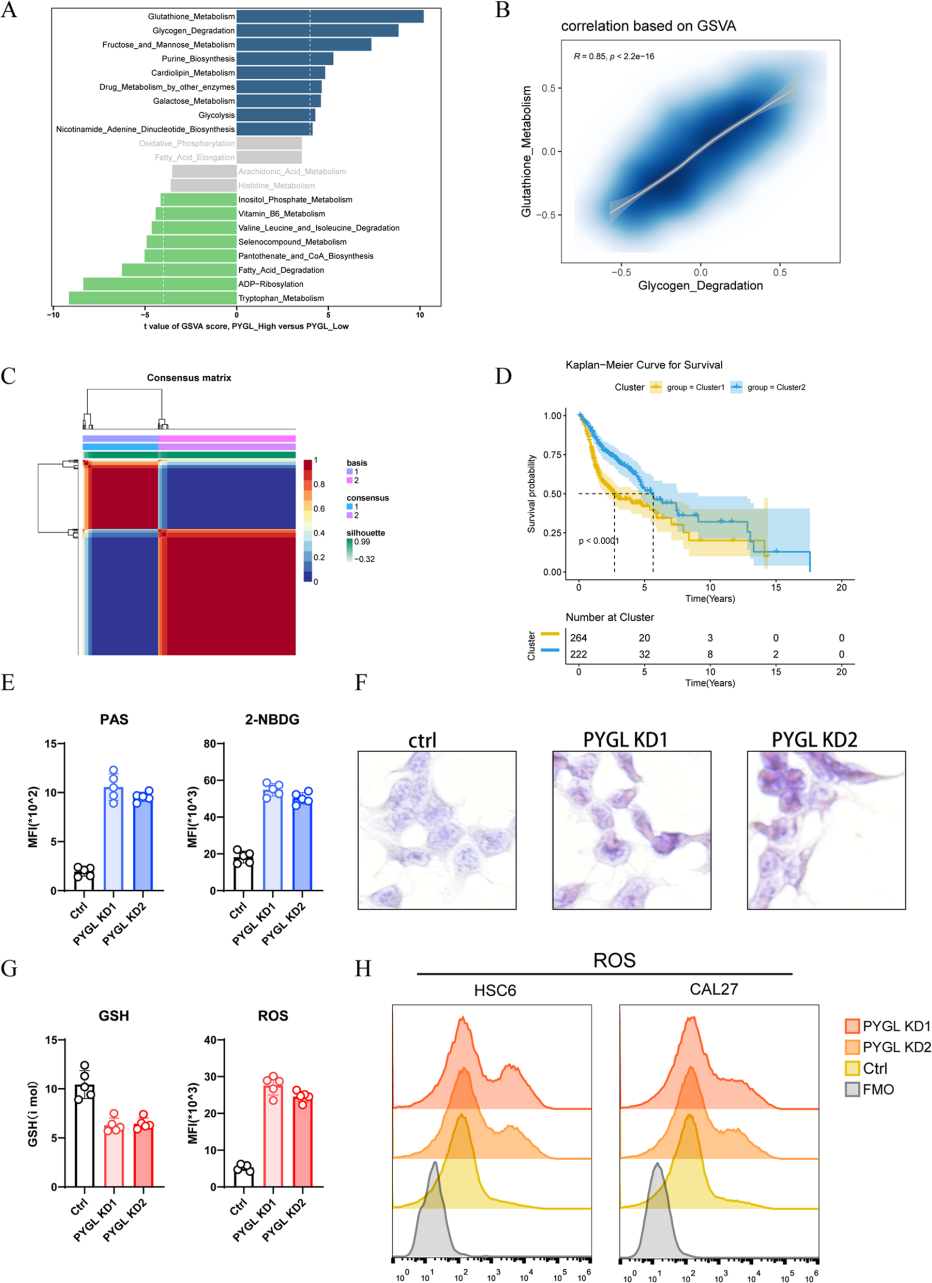
GSH metabolism correlated with *PYGL*-mediated glycogen degradation and poor prognosis. **A** GSVA between *PYGL*-low and *PYGL*-high group. **B** Correlation analysis between glycogen degradation and GSH metabolism. **C** Consensus map for NMF clustering. **D** Kaplan–Meier curves for survival probability of the two clusters. **E** The result of PAS and 2-NBDG assay in *PYGL* knock-down groups (*n* = 5). **F** Glycogen accumulation in *PYGL* knock-down groups was detected by standardized PAS staining. **G** The level of GSH and ROS in *PYGL* knock-down groups (*n* = 5). **H** ROS level was measured by ROS detection in *PYGL* knock-down groups

As mentioned earlier, METArisk phenotype related to cell metabolism reprogramming was potential to classify inter-patient heterogeneity and clinical outcomes in HNSCC; hence we next sought to identify the metabolic characteristics that attributed to poor prognosis and correlated with *PYGL*’s expression. Employing gene set variation analysis (GSVA), we examined the activated discrepancy of 113 defined metabolism related pathways in *PYGL*-high and *PYGL*-low subgroups. Apart from glycogen degradation mediated directly by *PYGL*, several pathways including GSH metabolism, fructose and mannose metabolism, purine biosynthesis and cardiolipin metabolism were also up-regulated in *PYGL*-high group (Fig. 7A), and correlation analysis of these enriched pathways (Fig.S8) showed that *PYGL*-mediated glycogen degradation was significantly associated with GSH metabolism, wherein targeted analysis confirmed the positive correlation of the two pathways (*R* = 0.85, *P* < 2.2e-16) (Fig. 7B). To further determine the correlation between *PYGL* and GSH metabolism, we conducted serious correlation analysis between *PYGL* and genes of GSH metabolism pathway, which demonstrated the positive correlations between *PYGL* and nine genes motivating GSH metabolism at transcriptional level (*R* > 0.5, *P* < 2.2e-16) (Fig.S9). *GSS* and *GCLM* express enzymes that synthesize GSH; *GSR*, *IDH1* and *G6PD* express enzymes promoting the transform progress from oxidized glutathione (GSSG) to GSH; *GSTA1*, *GSTM3* and *GSTM4* express the oxidoreductases of the redox reaction between GSH and peroxide; *GPX2* expresses invertase catalyzing the biochemical reaction from GSH to *GSSG*, which serves as a significant biological progress for cells to dispose the majority of ROS. Results above all indicated that *PYGL* has significant correlation with GSH metabolism pathway. To explore the role of GSH metabolism in HNSCC, NMF cluster analysis based on the genes of GSH metabolism pathway were applied and HNSCC cohort was categorized into GSH-active cluster and GSH-silent cluster according to the distinct activation pattern of the pathway (Fig. 7C). Based on these two clusters, a series of analyses were conducted to investigate the impact of GSH metabolism on HNSCC. By using ESTIMATE algorithm, a method evaluating tumor microenvironment, the lower ESTIMATE scores representing for inferior immune infiltration, and the higher tumor purity scores suggesting superior malignancy, were both observed in GSH-active cluster, indicating that GSH metabolism pathway is associated with HNSCC malignancy. (Fig.S10A and B). Moreover, Kaplan–Meier analysis on two clusters also illustrated the relationship between GSH metabolism and poor prognosis of patients with HNSCC, as GSH-active cluster exhibited worse clinical outcomes than GSH-silent cluster (Fig. 7D). These results represented that the activation of GSH metabolism performed similar effect on tumor malignancy as *PYGL* activation, and also could lead to poor prognosis, illustrating that GSH probably in the same regulation pathway with *PYGL* in HNSCC.

To further investigate the underlying regulatory relationship of *PYGL*-mediated glycogen degradation and GSH metabolism, we examined the variations of these two biochemical processes after *PYGL* knock-down. Standardized PAS staining and 2-NBDG detection revealed the increasing glycogen accumulation in *PYGL* knock-down groups (Fig. 7E and F), which indicated that glycogen degradation was blocked after the suppression of *PYGL*. Inhibition of glycogen degradation possibly led to reduction of glucose-6-phosphate (G6P) that was indispensable for the synthesis of GSH through pentose phosphate pathway, and decreasing GSH level was detected in *PYGL* knock-down groups (Fig. 7G). The consumption of ROS by GSH, an essential biological process maintaining the normal cells, was reported as an approach for malignant cells escaping the surveillance by *TP53* [32]. By conducting ROS detection, we observed the rise of ROS after *PYGL* knock-down (Fig. 7G and H), which illustrated that *PYGL* probably suppressed *TP53* through ROS consumption mediated by GSH metabolism and ultimately promoted the evolvement of HNSCC.

## Discussion

Cancer cells must react rapidly to internal and external stimuli to maintain high proliferation rates and survive in unfavorable environment with low oxygen, nutrition deficiency, and even chemotherapy drug intoxication. One way is to reprogram cell metabolism, thus affecting intra- and extra-cellular metabolites, which can have a major impact on gene expression, cellular differentiation and tumor microenvironment [9, 33]. Such metabolic reprogramming, especially in energy metabolism, has long been accepted as a hallmark and general phenomenon of tumors. For example, glycolysis has been found to significantly influence tumor development, metastasis and treatment susceptibility through a variety of biological processes [34]. Thus, the flexible and sophisticated metabolic network of cancer cells, as a hub for numerous cellular signaling pathways, may provide new therapeutic targets in the treatment of cancer [35]. Recent studies on metabolic reprogramming in HNSCC have provided promising potential targets in the treatment of HNSCC [36–38], demonstrating that comprehensively research on metabolic heterogeneity and relevant metabolic mechanisms is significantly demanded.

Primarily based on bulk RNA-seq, previous multi-patient sample transcriptomics studies on HNSCC mostly concentrated on screening prognostic genes [39] and predicting clinical outcomes [40], but with little concerns of intercellular heterogeneity. Simultaneously, single-cell sequencing studies on HNSCC mainly focused on functions of various cell component [41], discovery of new cell subsets [42], and investigation of intercellular heterogeneity [43], but were lack of the analyses between specific tumor cell subsets and patients prognosis for the limitation of small sample size. With combination of single-cell analysis and multi-patient sample sequencing, we firstly explored the relationship between specific cell subsets and prognosis in HNSCC, and HNSCC patients were classified from the perspective of unique metastasis-related malignant cell populations for further selection on therapeutic targets.

In our study, we first conducted single-cell RNA sequencing studies to investigate the metabolic heterogeneity in primary and metastatic samples from HNSCC patients. Combined with several functional analyses and AUCell algorithm, we revealed that the HNSCC metastases showed increased abundance of a specific cluster of malignant cells termed as META-active cells, which possessed high metabolic activity compared with the primary tumor. Analysis of patient-specific variation in hierarchy composition captured and integrated genomic profiles from single-cell and bulk RNA sequencing and clinical outcomes from large cohorts and functional properties of cancer metabolism within a single classification framework, which classified samples into METArisk-high group and METArisk-low group according to the proportion of METAactive malignant cells. METArisk-high group with high proportion of METAactive malignant cells showed lower overall survival, implying that metabolic heterogeneity might serve as a crucial factor affecting the prognosis of HNSCC. In order to figure out the biomarkers associated with the distinction of the hierarchy composition and clinical outcomes, we performed SVM-RFE and multivariate Cox regression analyses on the DEMRGs between the high and low METArisk groups. *PYGL* was finally selected as the key biomarker of our research, for it can lead to poorer prognosis, and was highly expressed both in tumor and METArisk-high group in transcriptional level, and also in HNSCC samples in translational level.

*PYGL*, located on chromosome 14q22.1 with 20 exons in total [44], is one of the genes related to hypoxia metabolism and was found to be up regulated in HNSCC [45]. *PYGL* is expressed as glycogen phosphorylase (GP), the key enzyme of glycogenolysis, which is responsible for the release of glycose-1-phosphate (G1P) from hepatic and muscle glycogen under physiological conditions [46]. In malignant cells, GP is involved in the glycogen catabolism and antioxidant defenses [47]. In previous studies, *PYGL* was reported as a gene signature derived from HNSCC, which defines the hypoxia ‘metagene’ [45]. It is also upregulated in several other cancers, such as seminoma, brain cancer and papillary renal cell carcinoma, as shown at oncomine website (https://www.oncomine.org/) [48]. Previous researches also reported that *PYGL* could promote metastasis in pancreatic cancer [49], while disruption of *PYGL* induces necroptosis [50]. Moreover, inhibitors of glycogen degradation metabolism regulated by *PYGL* has already been discovered like CP91149, CP320626 and Flavoperidol, but neither of them has been applied in HNSCC treatment [51–54], implicating a great potential of *PYGL* targeting therapy. Despite these findings, the underlying molecular mechanisms between *PYGL* and HNSCC are yet to be clarified. Thus, we combined significant metabolic signatures aforementioned and performed further research to identify possible pathways.

Cell apoptosis and nuclear DNA damage remarkably increased while cell proliferation ability and the potential of migration and invasion diminished in *PYGL* knock-down HNSCC cells, which illustrated that *PYGL* can promote the malignancy of HNSCC. Drug resistance analysis and xenograft tumor model supported that *PYGL* enhanced the resistance of HNSCC to cisplatin, leading to its progression and metastasis. It was reported that expression of *TP53* markedly enhanced the susceptibility to cisplatin and cisplatin-induced cell death in variable cancers [55–57], and we discovered that low *TP53* expression in HNSCC was closely associated with higher IC50 of cisplatin while *TP53* was proved to have negative correlation with *PYGL* in expression level. The following experiment confirmed the speculation that *PYGL* can promote HNSCC’s evolvement by suppressing *TP53*, which was functionally verified by the discovery that *PYGL* knock-down resulted in a higher proportion of cells in G0 phase and lower proportion of both G1/S/M and G2 phase.

Correlation analysis of enriched metabolic pathways based on GSVA illustrated that GSH metabolism was significantly correlated with glycogen degradation regulated by *PYGL*, which was simultaneously confirmed at the transcriptional level. NMF and ESTIMATE analysis further revealed the activation of GSH metabolism pathway was closely related to higher tumor malignance and poorer prognosis. GSH metabolism on tumor has been discussed in previous research and was certified to affect tumor in different ways. As an important antioxidant in organisms, GSH expends ROS to control oxidative stress in tumor and promote tumor development, which was demonstrated in breast cancer [58], prostate cancer [59], and liver cancer [60]. GSH can also affect the apoptotic process by regulating the anti-apoptotic proteins and caspase activity of the Bcl-2 family, which was certified in breast cancer [61]. Ferroptosis was detected to be suppressed by GSH simultaneously in liver cancer [62] and triple-negative breast cancer [63]. Additionally, GSH was proved to enhance cisplatin resistance of tumor by several pathways [64]. However, the mechanism of GSH on HNSCC was still not clearly determined. Here, we demonstrated that *PYGL* can promote HNSCC’s evolvement by activating GSH metabolism and downstream pathway. *PYGL* catalyzes glycogen phosphorylation, and the degradation metabolite G1P transforms into G6P, which produces NADPH from pentose phosphate pathway. With the participation of NADPH, GSH reductase (GR) can reduce GSSG to GSH [65], decreasing ROS in tumor cells. Previous researches proved cell death by the activation of p53 was closely correlated with the accumulation of cellular ROS [66, 67]. Besides, the reduction of ROS can suppress p53’s function and promote the progression of tumor [68, 69]. To sum up, *PYGL* activates GSH metabolism to reduce ROS level, which suppresses the function of *TP53*, and ultimately promotes the evolvement and decrease the chemosensitivity of HNSCC.

There are also some limitations in our study. For instance, single-cell samples can be further expanded to reduce selection bias and ensure the randomness. It’s also a better consideration to generalize our model for studies on other cancer, which can not only demonstrate its universality but also expand the study for excavating the metabolic pathways. Furthermore, generally recognized as a complex network, tumor development can be affected by multiple factors via the GSH/ROS/p53 pathway. *PYGL* knock-down is bound to change the expression of other molecules, which probably affects the function of p53 and thus influences the long-term efficacy. Finally, we were not able to collect and adjust all the potential confounding factors in our Kaplan–Meier analysis, which may cause bias in our survival analysis. In addition to our current discoveries, further systemic studies are demanded to clarify relational molecular mechanism and conduct corresponding clinical studies in the future.

## Conclusion

**Fig.8:**
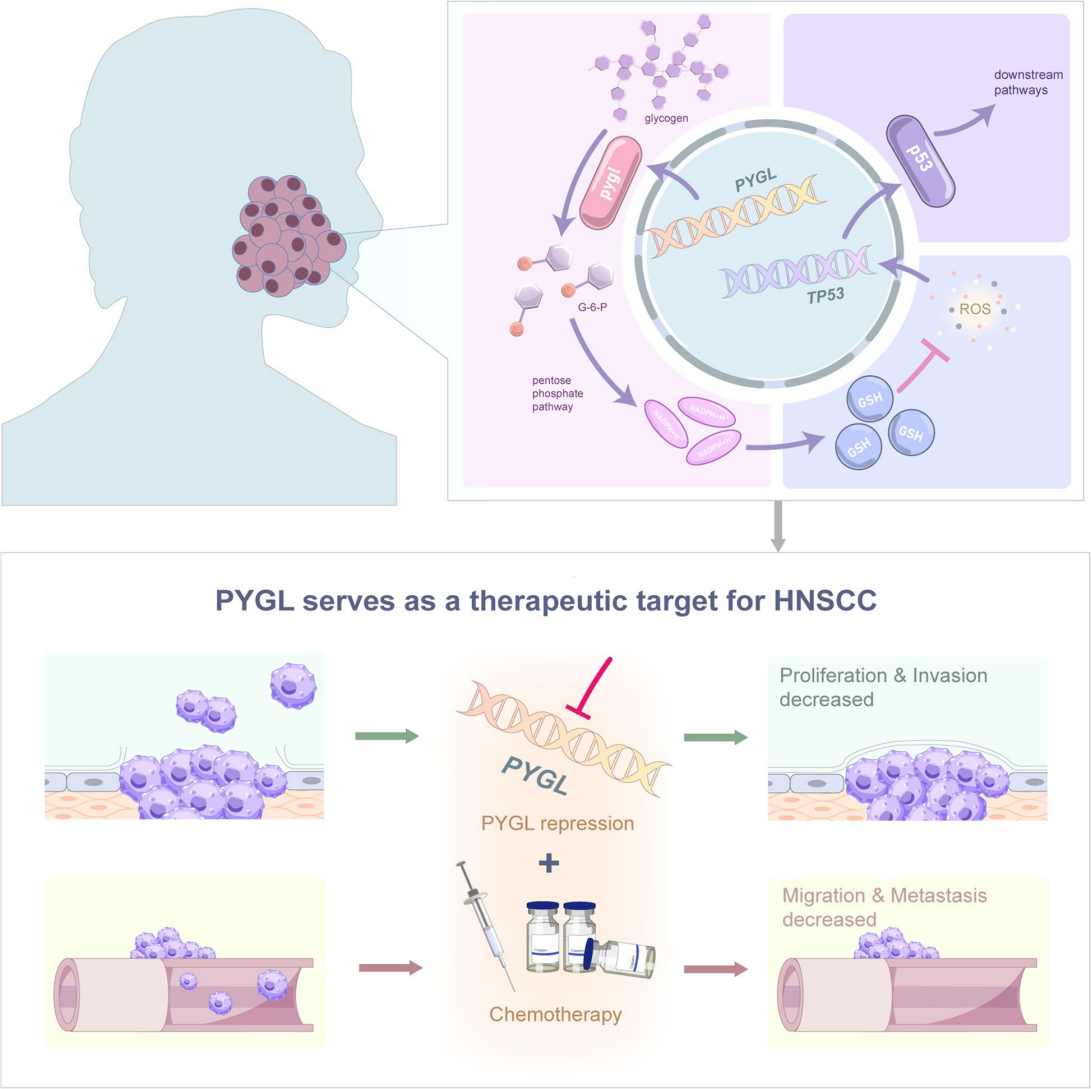
*PYGL* can serve as a therapeutic target for HNSCC

Collectively, our study revealed cell metabolism reprogramming was a crucial risk factor for HNSCC through the discovery of a unique cluster of malignant cells with high metabolic activity in metastatic samples and the construction of METArisk phenotype which illustrated the composition of each patient’s cell hierarchy and correlated with the poor prognosis. Furthermore, analyses of the DEMRGs in METArisk phenotypes suggested *PYGL*, the key biomarker in glycogen degradation, was strongly potential to guide the development and drug resistance of HNSCC by the *PYGL*/GSH/ROS/p53 pathway, thereby setting the foundation for new clinical therapies of HNSCC in the future (Fig. 8).

## Supplementary Information

**Additional file 1: Figure. S1**. A flowchart summarizing the study.  **Figure S2.** Distinct cellular construction of primary and metastatic samples. **Figure S3.** Cellular hierarchy of malignant cell including nine clusters. **Figure S4.** Volcano plot showing expression of DEGs (|logFC|>1 and adjusted *P* <0.05)between primary- and metastatic-specific populations. **Figure S5.** Distribution of AUC score and METArisk score. **Figure S6.** A total of 69 kinds of prediction models and further calculated the C-index of each model across validation datasets. **Figure S7.** Boxplot depicted the expression level in tumor and normal groups. **Figure S8.** Correlation analysis of pathways selected by GSVA. **Figure S9.** Pearson correlation analysis between PYGL and functional genes in GSH metabolism. **Figure S10.** ESTIMATE analysis illustrating ESTIMATE score (A) and tumor purity score (B) of GSH-active and GSH-silence cluster.

## Data Availability

The dataset supporting the conclusions of this article is available from the corresponding author upon request, and associated source codes is provided in https://github.com/Mcdull8/METArisk.

## Abbreviations

HNSCC: Head and neck squamous cell carcinoma
SRA: Sequence Read Archive
TCGA: The Cancer Genome Atlas
UMAP: Uniform manifold approximation and projection
DEGs: Differential expressed genes
KEGG: Kyoto Encyclopedia of Genes and Genomes
MRGs: Metabolism-Related Genes
GSEA: Gene Set Enrichment Analysis
GO: Gene Ontology
MSigDB: Molecular Signatures Database
AUC: Area under the curve
TPM: Transcripts per million
DEMRGs: Differential Expressed Metabolism-Related Genes
SVM-RFE: Support vector machine recursive feature elimination
IHC: Immunohistochemistry
HPA: Human Protein Atlas
CARE: Computational Analysis of Resistance
NMF: Non-negative matrix factorization
GSH: Glutathione
ESTIMATE: Estimation of Stromal and Immune cells in Malignant Tumor tissues using Expression
DMEM: Dulbecco's modified Eagle's medium
FBS: Fetal bovine serum
CCK-8: Cell Counting Kit-8
OD: Optical density
7-AAD: 7-aminoactinomycin D
DAPI: 4’,6-diamidino-2-phenylindole
ROS: Reactive oxygen species
DHE: Dihydroethidium
2-NBDG: 2-N-7-nitrobenz-2-oxa-1,3-diazol-4-yl amino-2-deoxyglucose
SDS-PAGE: Sodium dodecyl sulphate–polyacrylamide gel electrophoresis
PVDF: Polyvinylidene fluoride
ECL: Enhanced Chemiluminescence
GAPDH: Glyceraldehyde 3-phosphate dehydrogenase
PAS: Periodic acid Schiff
DTNB: 5,5′-dithiobis-2-nitrobenzoic acid
GSVA: Gene set variation analysis
GSSG: Oxidized glutathione
G6P: Glucose-6-phosphate
GP: Glycogen phosphorylase
G1P: Glycose-1-phosphate
