# Correction: Cellular hierarchy framework based on single-cell/multi-patient sample sequencing reveals metabolic biomarker PYGL as a therapeutic target for HNSCC

**DOI:** 10.1186/s13046-025-03448-x

**Published:** 2025-07-03

**Authors:** Jiezhong Guan, Xi Xu, Guo Qiu, Chong He, Xiaoyue Lu, Kang Wang, Xinyu Liu, Yuanyuan Li, Zihang Ling, Xuan Tang, Yujie Liang, Xiaoan Tao, Bin Cheng, Bo Yang

**Affiliations:** 1 Hospital of Stomatology, Guanghua School of Stomatology, Sun Yat-Sen University, Guangdong Provincial Key Laboratory of Stomatology, Guangzhou, China; 2 Department of Hematology, Nanfang Hospital, Southern Medical University, Guangzhou, China; 3 Zhongshan School of Medicine, Sun Yat-Sen University, Guangzhou, China

**Correction: J Exp Clin Cancer Res 42, 162 (2023)**

**https://doi.org/10.1186/s13046-023-02734-w**

Following the publication of the original article [1], the authors identified error in Fig. 5F where the metastatic nodules in mice after PYGL-KD and cisplatin therapy, was mistakenly displayed.

The correct figure is presented below:

**Incorrect **Fig. 5f

**Fig.5:**
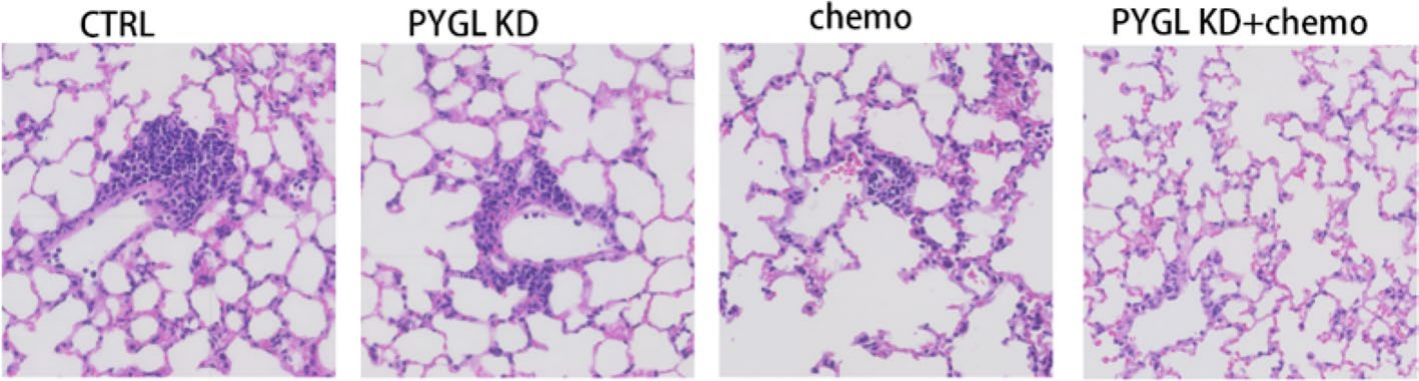
Chemotherapy resistance and metastasis of HNSCC promoted by *PYGL*. **A** Prediction of cisplatin IC50 in METArisk-high and METArisk-low groups. **B** Prediction of cisplatin IC50 in high and low *PYGL* expression groups. **C** Computational analysis of resistance with *PYGL* of cisplatin in the CARE database. **D** Tumor growth was measured once a week in tumor-bearing nude mice stably transfected with HNSCC cells (*n* = 5). **E** The tumors were removed, and the tumor weight was analyzed after the mice were executed. **F** Metastasis of HNSCC was measured by the results of hematoxylin–eosin staining of mice lung tissue

**Correct **Fig. 5f

**Fig.5:**
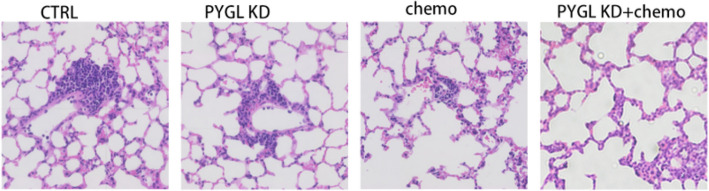
Chemotherapy resistance and metastasis of HNSCC promoted by *PYGL*. **A** Prediction of cisplatin IC50 in METArisk-high and METArisk-low groups. **B** Prediction of cisplatin IC50 in high and low *PYGL* expression groups. **C** Computational analysis of resistance with *PYGL* of cisplatin in the CARE database. **D** Tumor growth was measured once a week in tumor-bearing nude mice stably transfected with HNSCC cells (*n* = 5). **E** The tumors were removed, and the tumor weight was analyzed after the mice were executed. **F** Metastasis of HNSCC was measured by the results of hematoxylin–eosin staining of mice lung tissue

The correction does not compromise the validity of the conclusions and the overall content of the article. The original article [1] has been updated.
