# Supplementary material for: Cellular hierarchy framework based on single-cell/multi-patient sample sequencing reveals metabolic biomarker PYGL as a therapeutic target for HNSCC
Source: J Exp Clin Cancer Res. 2023 Jul 8;42:162. doi: 10.1186/s13046-023-02734-w (PMC10329320; doi:10.1186/s13046-023-02734-w)
Supplement: Supplementary file 1 — Additional file 1: Figure. S1. A flowchart summarizing the study. Figure S2. Distinct cellular construction of primary and metastatic samples. Figure S3. Cellular hierarchy of malignant cell including nine clusters. Figure S4. Volcano plot showing expression of DEGs (|logFC|>1 and adjusted P <0.05)between primary- and metastatic-specific populations. Figure S5. Distribution of AUC score and METArisk score. Figure S6. A total of 69 kinds of prediction models and further calculated the C-index of each model across validation datasets. Figure S7. Boxplot depicted the expression level in tumor and normal groups. Figure S8. Correlation analysis of pathways selected by GSVA. Figure S9. Pearson correlation analysis between PYGL and functional genes in GSH metabolism. Figure S10. ESTIMATE analysis illustrating ESTIMATE score (A) and tumor purity score (B) of GSH-active and GSH-silence cluster. [file 13046_2023_2734_MOESM1_ESM.docx]

**SUPPLEMENTARY MATERIAL**


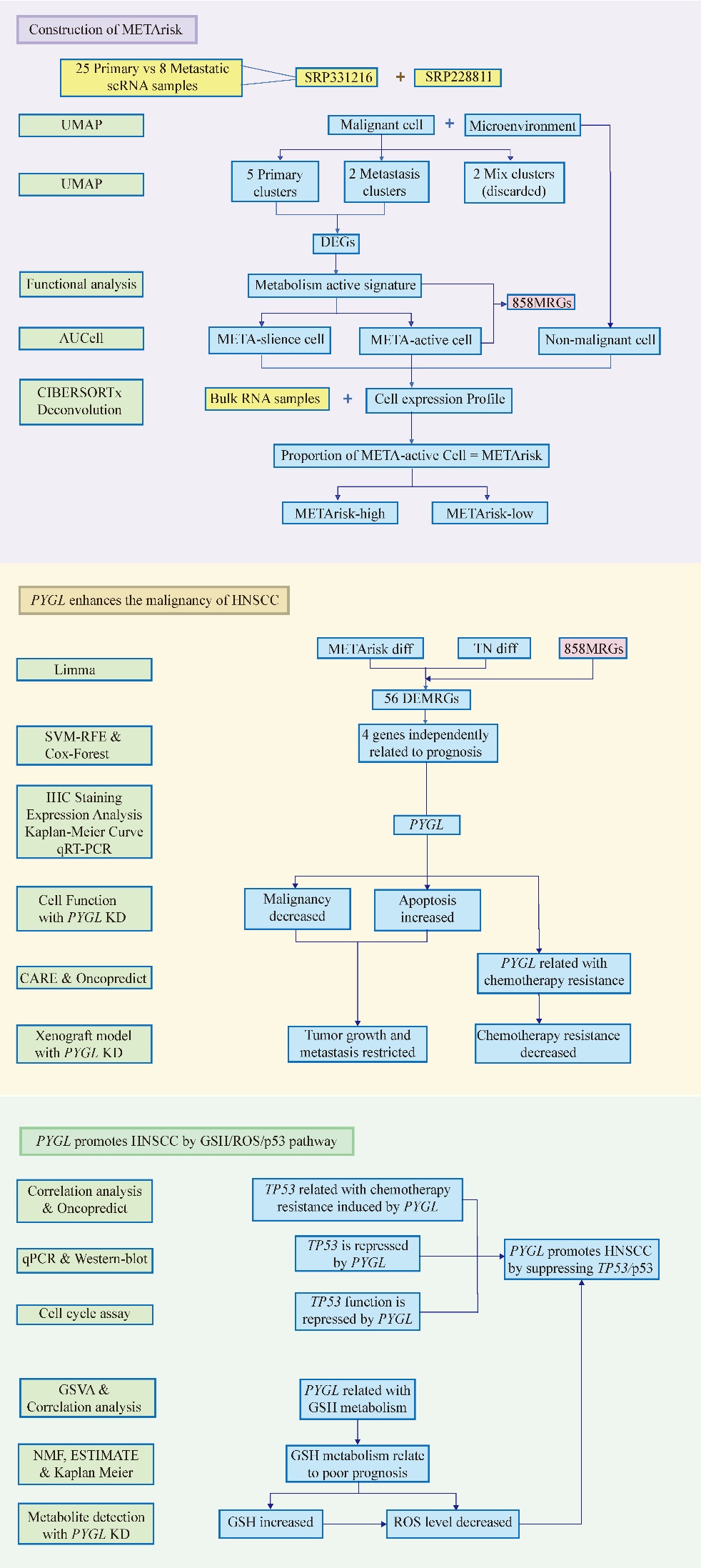


**Figure S1.** A flowchart summarizing the study.


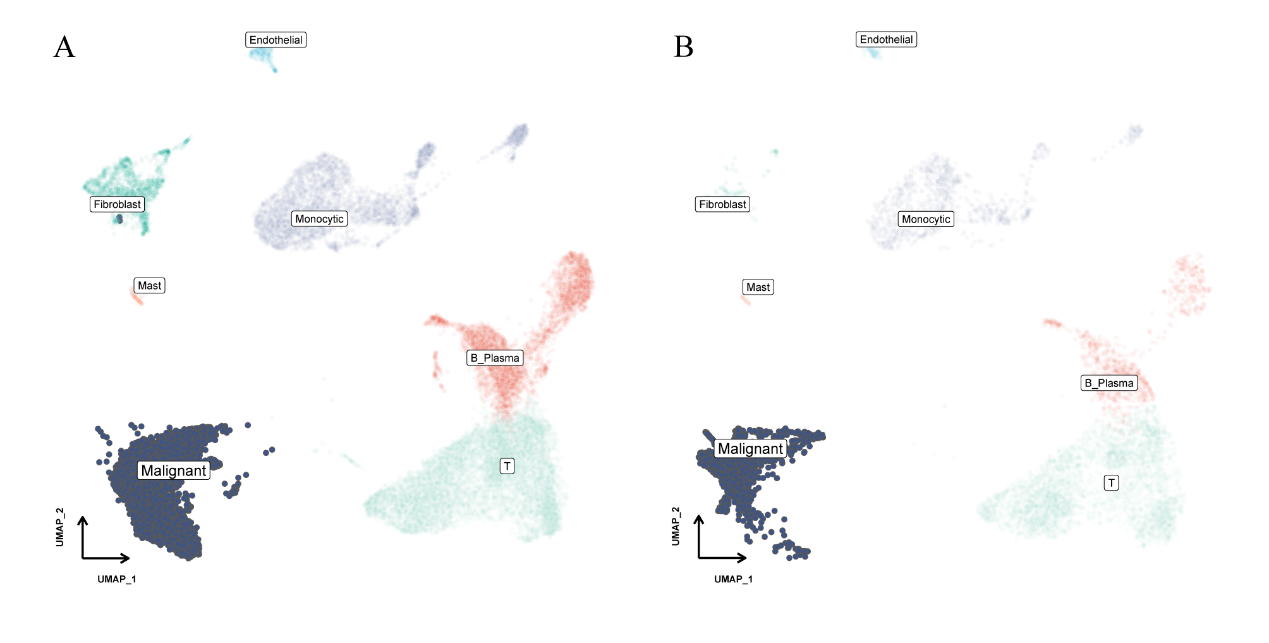


**Figure S2. Distinct cellular construction of primary and metastatic samples.**

(A) Diffusion map of cells from primary samples, colored by cell-type annotation. (B) Diffusion map of cells from metastatic samples, colored by cell-type annotation.


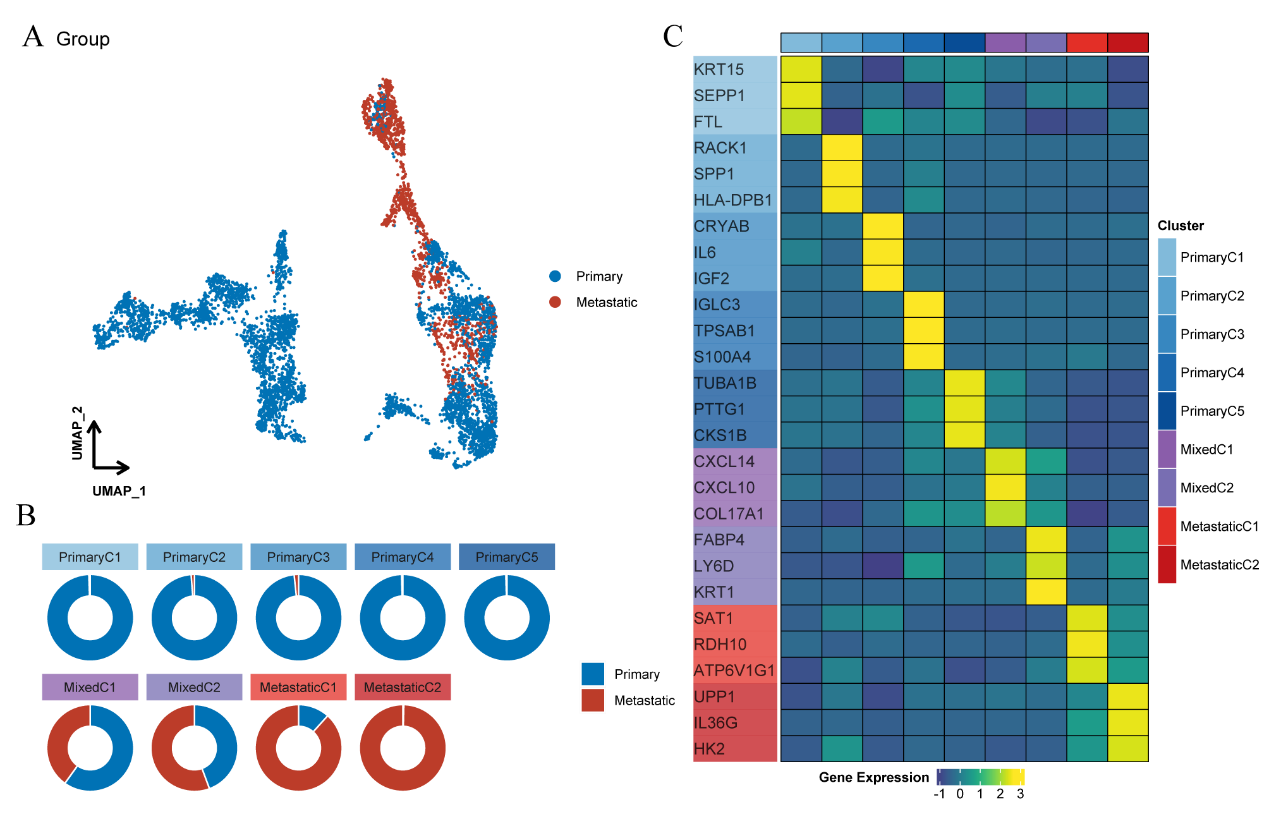


**Figure S3. Cellular hierarchy of malignant cell including nine clusters.**

(A) Diffusion map of malignant cells extracted and re-clustered, colored by sample origin. (B) Detailed cell proportion of each cluster in Fig.1C. (C) Marker genes of each cluster.


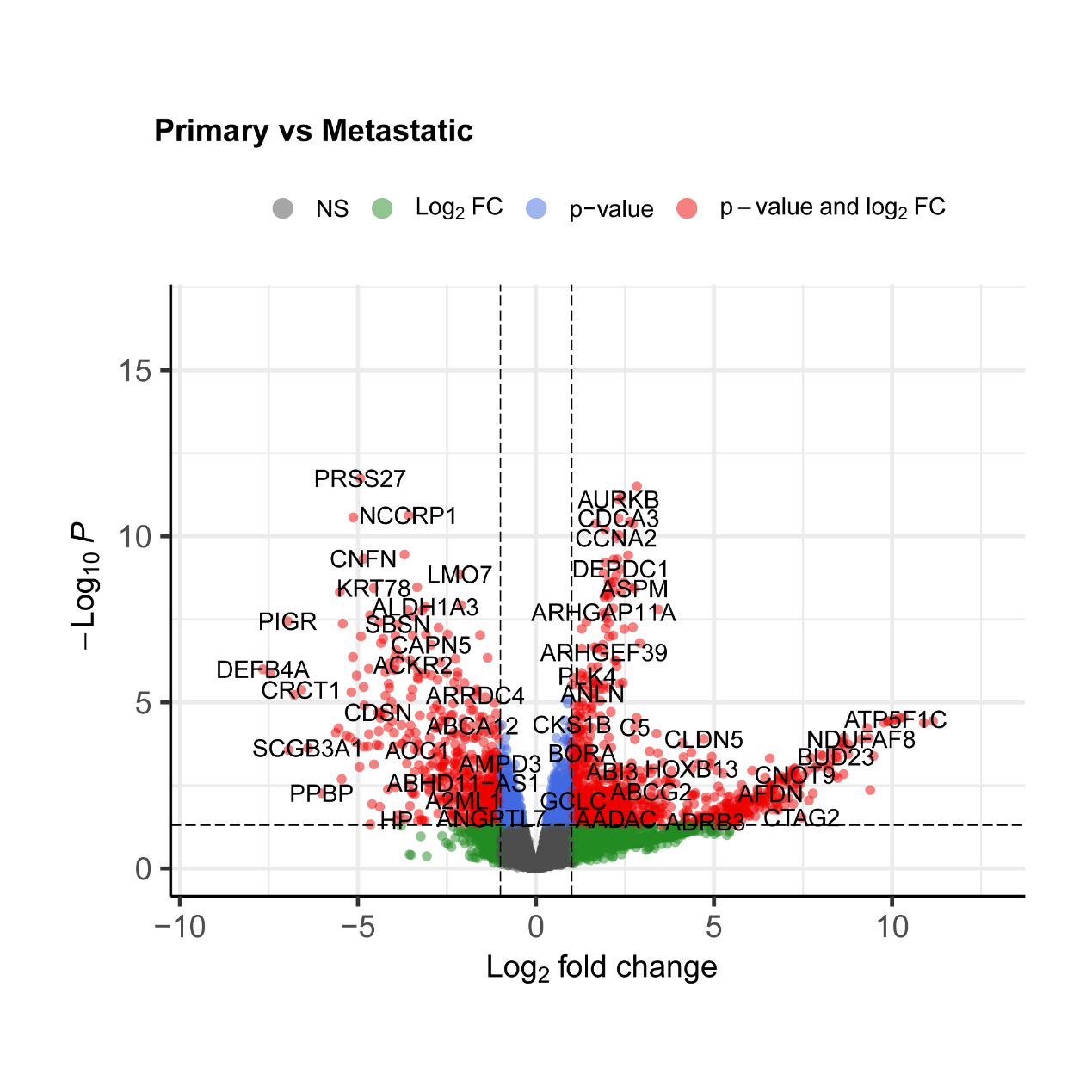


**Figure S4.** Volcano plot showing expression of DEGs (|logFC|>1 and adjusted *P* <0.05) between primary- and metastatic-specific populations.


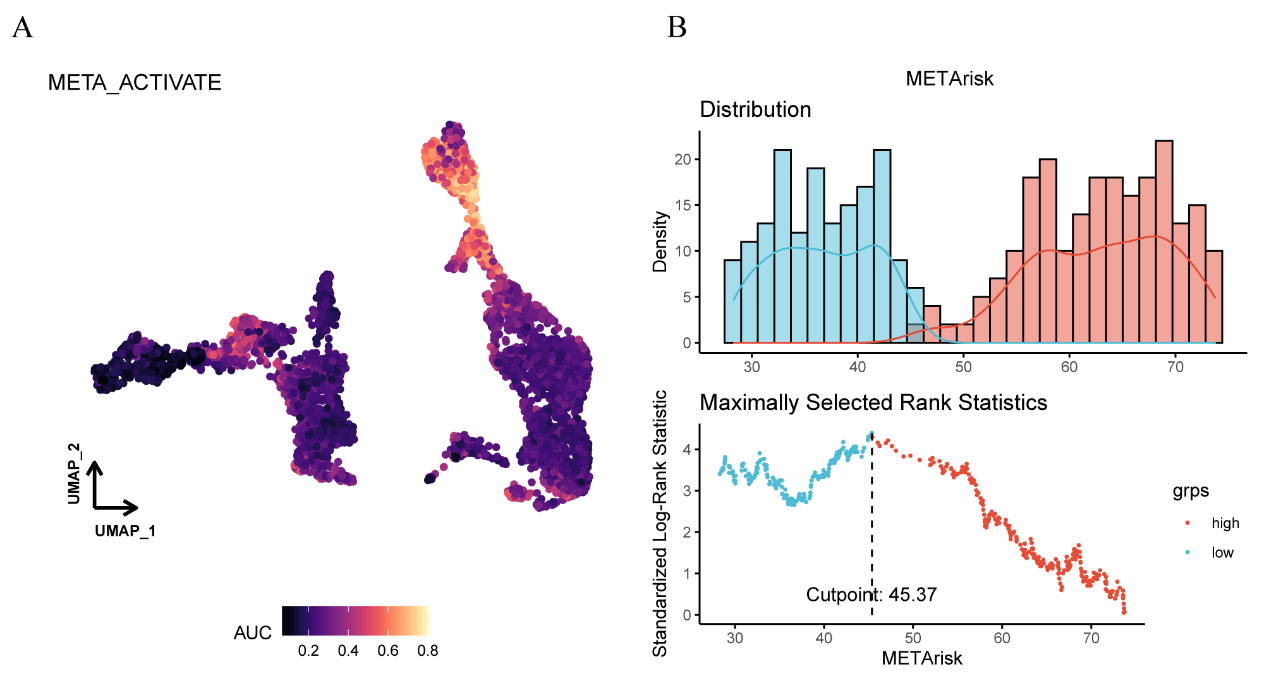


**Figure S5. Distribution of AUC score and METArisk score**

(A) UMAP plot based on the AUC score of each cell. Clusters with high AUC score are highlighted. (B) The optimal cutoff according to maximally selected rank statistics divided patients into METArisk-high group and METArisk-low group.


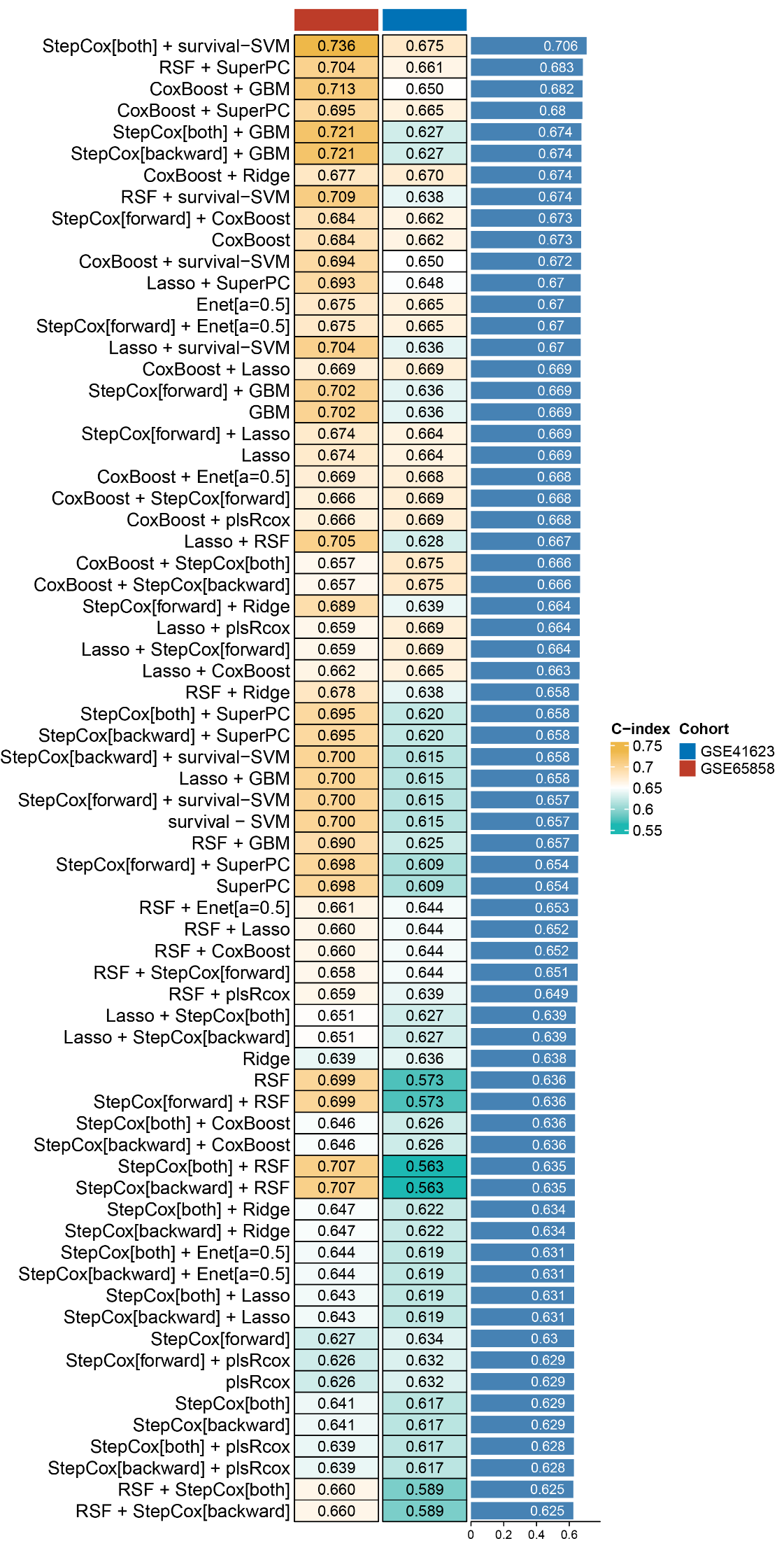


**Figure S6.** A total of 69 kinds of prediction models and further calculated the C-index of each model across validation datasets.


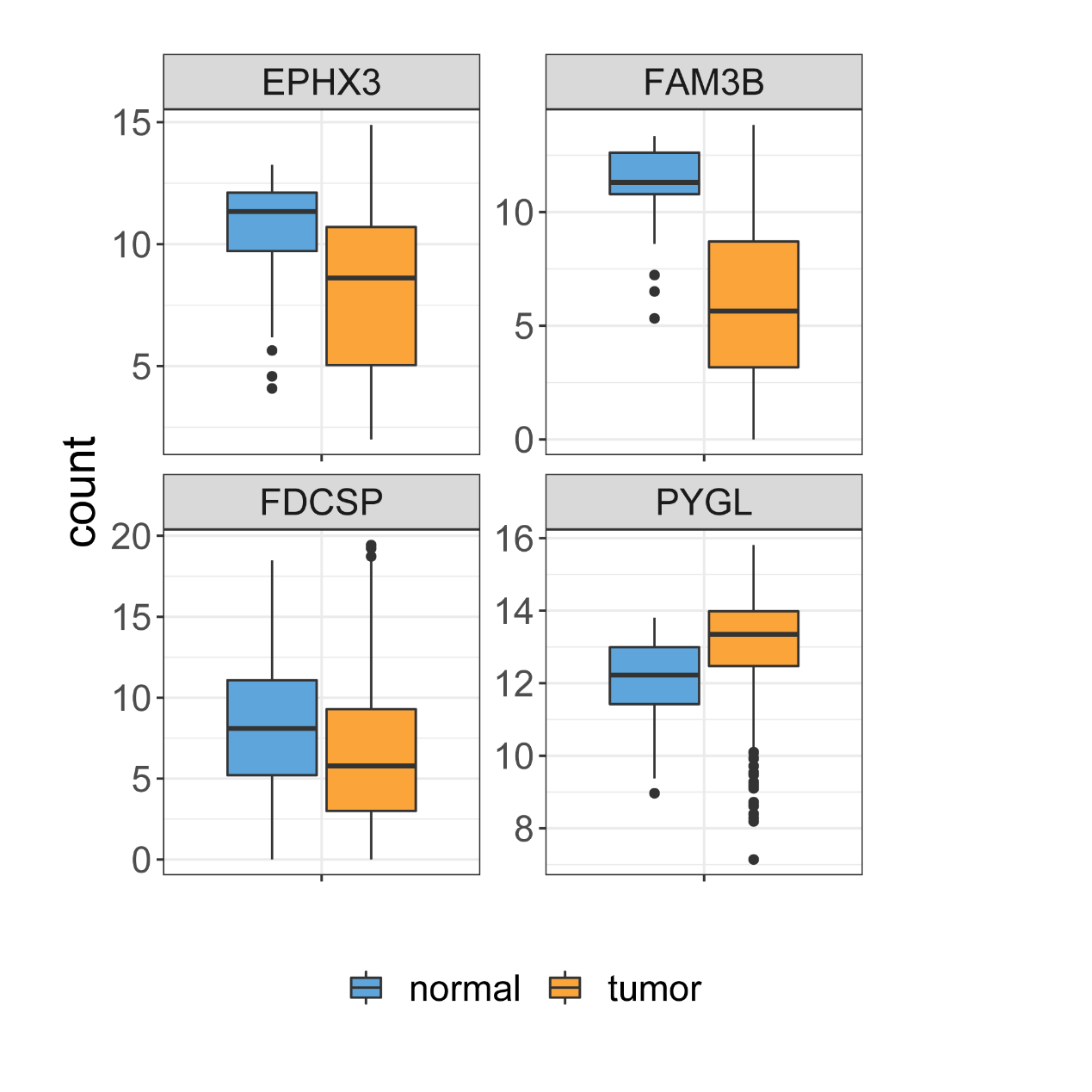


**Figure S7.** Boxplot depicted the expression level in tumor and normal groups.


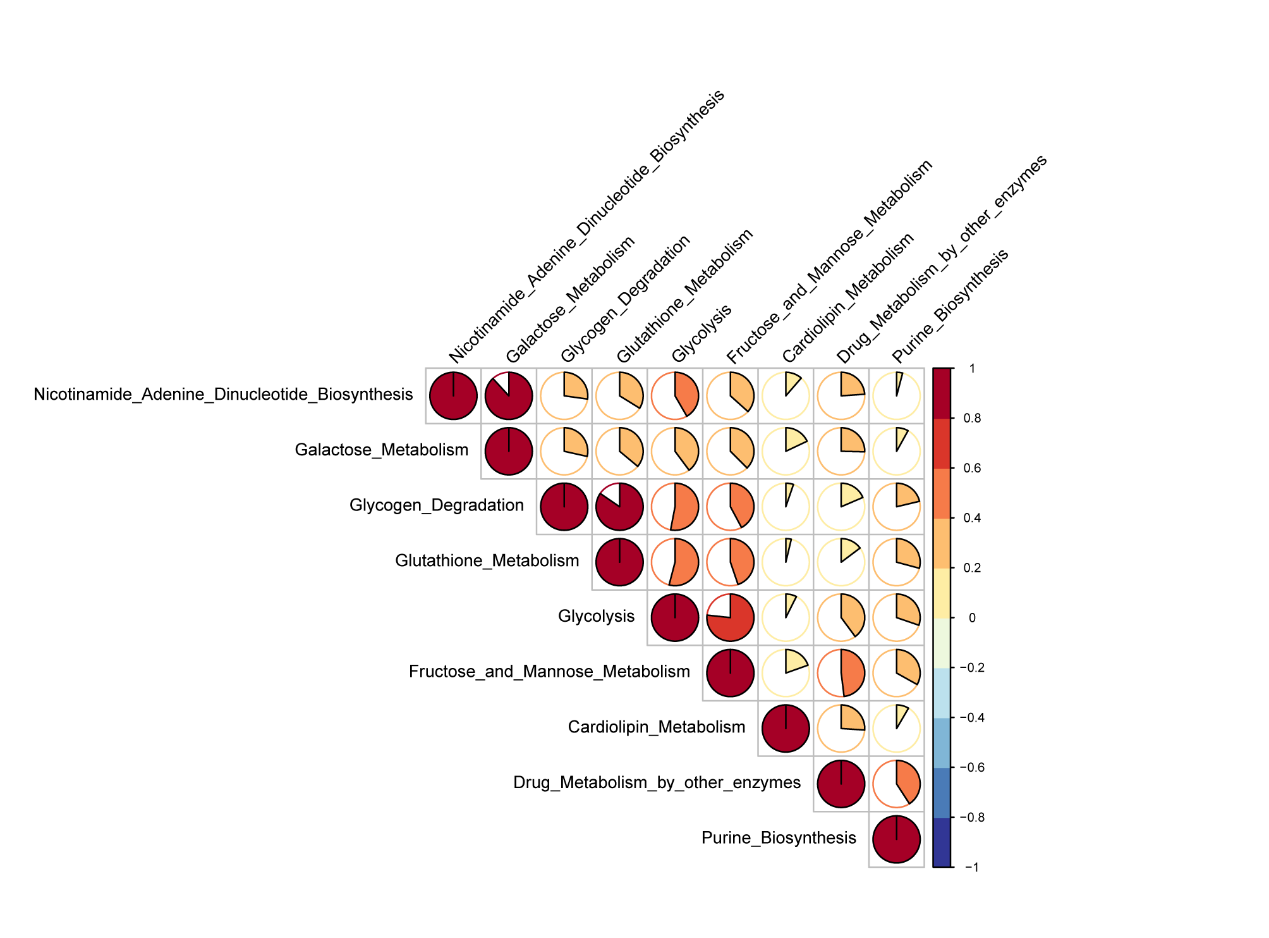


**Figure S8.** Correlation analysis of pathways selected by GSVA.


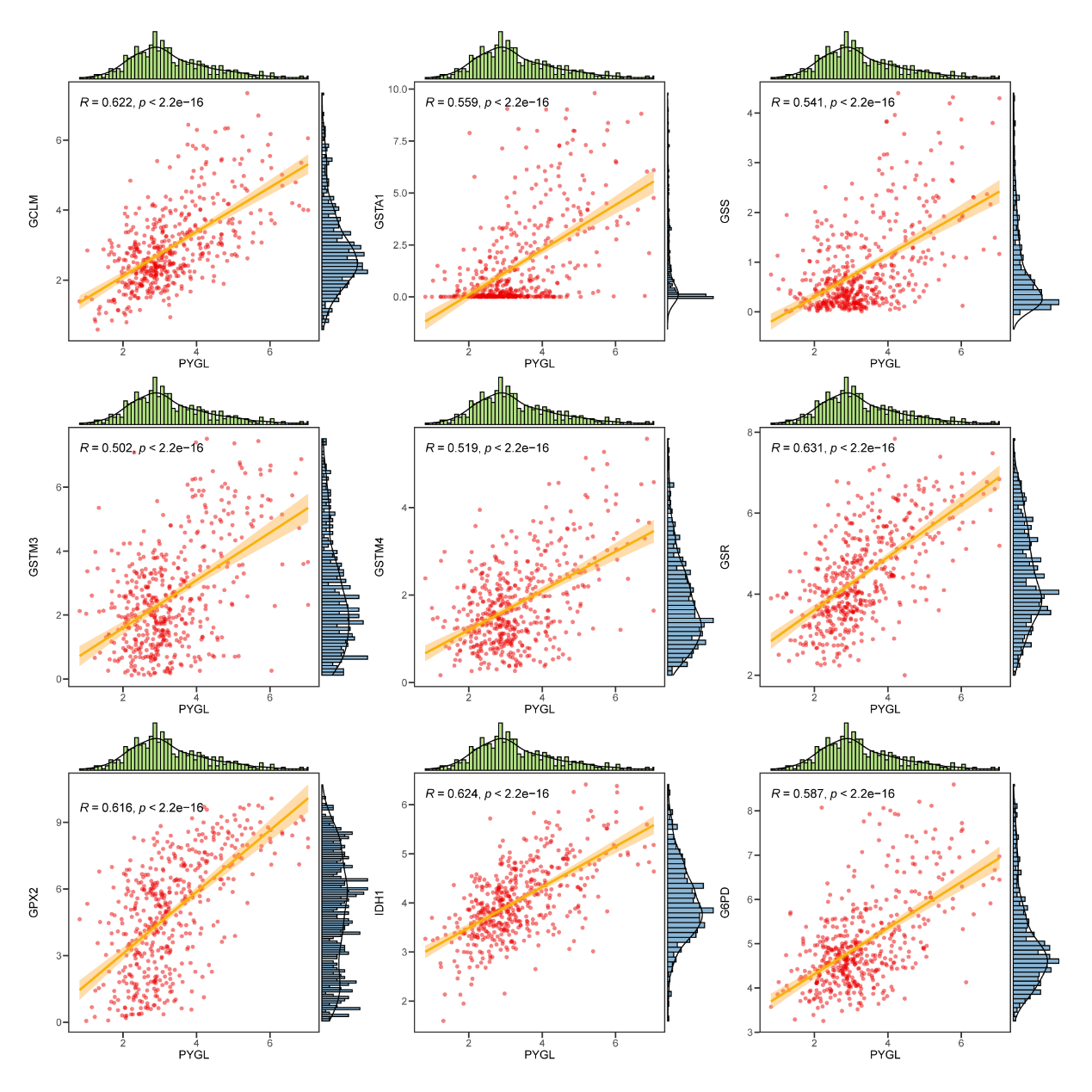


**Figure S9.** Pearson correlation analysis between *PYGL* and functional genes in GSH metabolism.


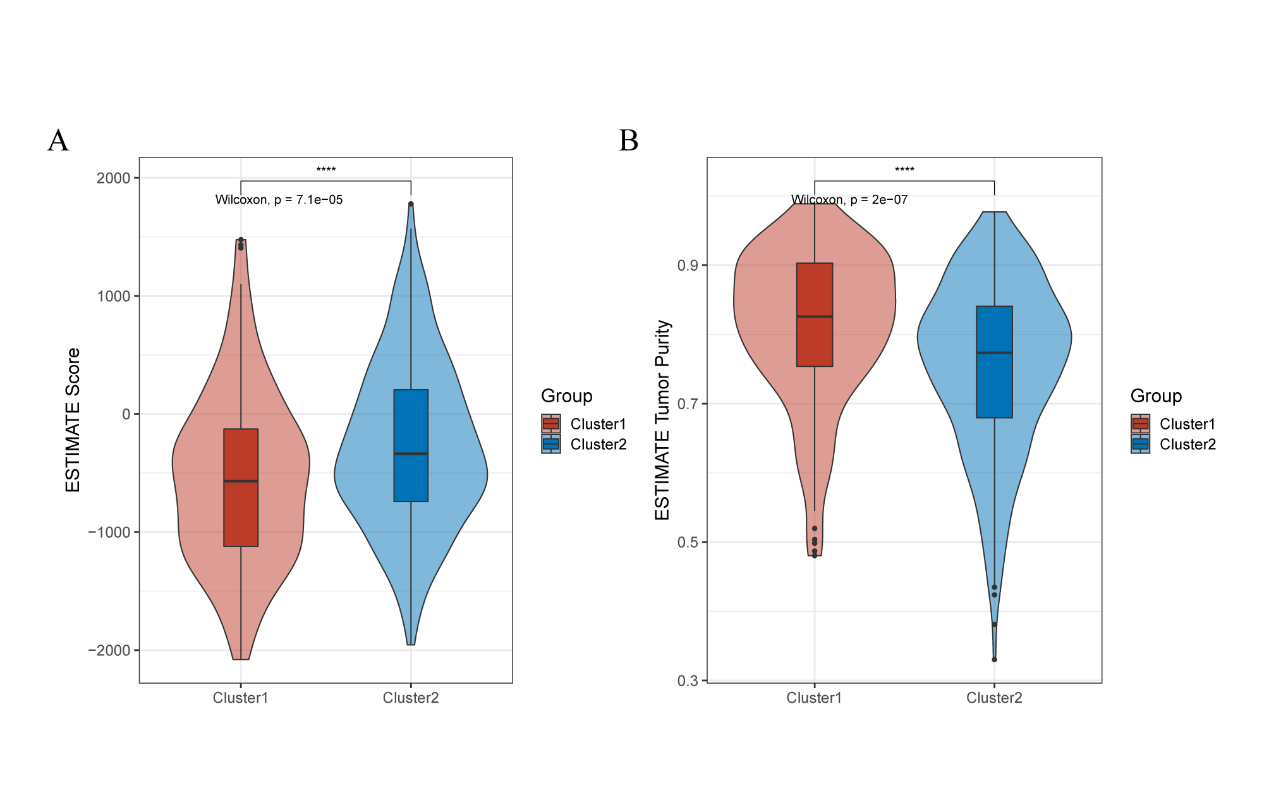


**Figure S10.** ESTIMATE analysis illustrating ESTIMATE score (A) and tumor purity score (B) of GSH-active and GSH-silence cluster.
